# Promising Non-cytotoxic Monosubstituted Chalcones to Target Monoamine Oxidase-B

**DOI:** 10.1021/acsmedchemlett.1c00238

**Published:** 2021-06-14

**Authors:** Luca G. Iacovino, Luca Pinzi, Giorgio Facchetti, Beatrice Bortolini, Michael S. Christodoulou, Claudia Binda, Giulio Rastelli, Isabella Rimoldi, Daniele Passarella, Maria Luisa Di Paolo, Lisa Dalla Via

**Affiliations:** † Dipartimento di Biologia e Biotecnologie, Università di Pavia, Pavia 27100, Italy; ‡ Dipartimento di Scienze della Vita, Università degli Studi di Modena e Reggio Emilia, Modena 41125, Italy; § DISFARM, Sezione di Chimica Generale e Organica “A. Marchesini”, Università degli Studi di Milano, Milano 20133, Italy; ∥ Dipartimento di Scienze del Farmaco, Università degli Studi di Padova, Padova 35131, Italy; ⊥ Dipartimento di Chimica, Università degli Studi di Milano, Milano 20133, Italy; # Dipartimento di Medicina Molecolare, Università degli Studi di Padova, Padova 35131, Italy

**Keywords:** Chalcones, cytotoxicity, monoamine oxidase, molecular modeling, crystallographic analysis

## Abstract

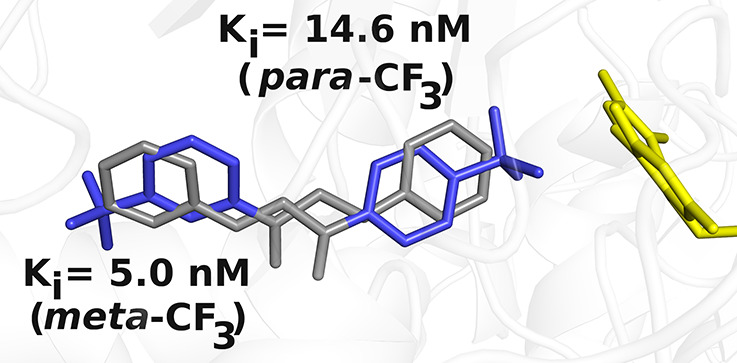

A library of monosubstituted chalcones (**1**–**17**) bearing electron-donating and electron-withdrawing groups on both aromatic rings were selected. The cell viability on human tumor cell lines was evaluated first. The compounds unable to induce detectable cytotoxicity (**1**, **13**, and **14**) were tested using the monoamine oxidase (MAO) activity assay. Interestingly, they inhibit MAO-B, acting as competitive inhibitors, with **13** and **14** showing the best profiles. In particular, **13** exhibited a potency higher than that of safinamide, taken as a reference. Docking studies and crystallographic analysis showed that in human MAO-B **13** binds with the halogen-substituted aromatic ring in the entrance cavity, similar to safinamide, whereas **14** is accommodated in the opposite way. The main conclusion of this cell biology, biochemistry, and structural study is to highlights **13** as a chalcone derivative that is worth consideration for the development of novel MAO-B-selective inhibitors for the treatment of neurodegenerative diseases.

Chalcones are a key structure motif within the kingdom Plantae and are widely found in many edible plants.^1^ They are open-chain flavonoids in which a three-carbon α,β-unsaturated carbonyl moiety joins the two aromatic rings and are considered as open-chain intermediates in the synthesis of flavones.^2^ This peculiar chemical structure endows chalcones with the capability to hit different targets (enzymes and receptors) and to exert a variety of biological activities. The chalcone skeleton is therefore widely used in medicinal chemistry and drug discovery^3,4^ to obtain derivatives with anticancer, antioxidant, antiviral, and anti-inflammatory activities and for the treatment of neurodegenerative disorders.^5−9^

Chalcone derivatives exert their anticancer activity through multiple mechanisms involving different targets,^8,10^ and various targets are also responsible for the effect of chalcone analogues on neurodegenerative diseases.^11−13^ In this latter field, monoamine oxidases (MAOs) play an important role, being mitochondrial and flavin adenine dinucleotide (FAD) cofactor-dependent enzymes, which catalyze the oxidative deamination of monoamine neurotransmitters.^14^ In particular, the MAO-B isoform is mainly investigated for treatments of neurodegenerative disorders (including Parkinson’s and Alzheimer’s diseases), while the MAO-A isoform mostly deals with neurological and psychiatric disorders.^14−16^ In this connection, although a number of MAO-A and MAO-B inhibitors are already available in clinical practice, the need to reduce their side effects and/or to improve their selectivity is still definitely of interest and encourages studies aimed at developing novel types of inhibitors.

The chalcone structure has been considered an interesting non-nitrogen-containing scaffold.^17−20^ In a recent paper, detailed structure–activity relationships (SAR) were discussed for the inhibitory activity and selectivity toward MAOs, with regard to the influence of different substituents on both aromatic rings of chalcone derivatives and compounds containing the chalcone motif.^17^ In particular, the introduction of heterocycles, the presence of nitro groups or lipophilic electron-donating groups, and halogen substitution on phenyl ring A and/or B are some of the modifications that were designed for the chalcone scaffold, and frequently, more than one modification/substitution was introduced on the same compound.^17^ On the basis of literature data, among the simplest, less substituted chalcone derivatives, the most active compounds against the MAO-B isoform are characterized by the presence of hydroxyl and methoxy substituents at *ortho* and *para* positions, respectively, of the A aromatic ring and a chlorine atom at the *para* position of the B ring. Nevertheless, a role for each specific substituent did not emerge.^12,13,17,18,21^

In this frame, we focused our attention on a small library of monosubstituted chalcone derivatives bearing an electron-donating or electron-withdrawing substituent in the A or B ring, with the aim to further clarify the function of the single substituent in the biological properties. Such derivatives were previously reported and studied as antiproliferative agents on leukemia cells,^22^ and for them few or no data on MAO activity were reported.^19,23^ All of the compounds were synthesized using a classical Claisen–Schmidt condensation of the appropriate acetophenone with the corresponding aldehyde in a methanolic solution of sodium hydroxide. The products were obtained after being recrystallized twice from ethanol with a good grade of purity (see the Supporting Information). As the absence of cytotoxicity is a mandatory requirement for compounds to be used to treat neurodegenerative diseases, the potential antiproliferative effects of the compounds belonging to the library were evaluated on a wider number of human tumor cell lines. The chalcone derivatives that were unable to induce any significant antiproliferative effect were selected and further studied as potential inhibitors for human recombinant MAO-A and MAO-B. In detail, we evaluated the inhibition constant values, the selectivity indices, and the mechanism of inhibition. The kinetic characterization of the most interesting derivatives, **13** and **14**, integrated with docking studies and crystallographic experiments uncovered their binding mode inside the MAO-B active site. Overall, our findings expand the knowledge in terms of possible molecular interactions between the chalcone scaffold and MAO-B and highlight **13** as a scaffold worth further study in view of the development of new promising drugs for neurodegenerative diseases.

The absence of any cytotoxicity and the enzyme inhibition mechanism are fundamental elements in the development of novel MAO-B inhibitors to target neurodegenerative diseases. With this aim, cell biology, biochemical, and structural studies were combined to explore the activity profiles of a small library of monosubstituted chalcones as potential MAO-B inhibitors.

Table 1 reports the chalcone series and the results on antiproliferative activity, which was evaluated through an *in vitro* assay performed on three tumor cell lines, *i.e.*, A2780 (ovarian carcinoma), HT-29 (colorectal adenocarcinoma), and MSTO-211H (biphasic mesothelioma). Data are expressed in terms of the GI_50_ value, *i.e.*, the concentration of compound able to inhibit 50% cell growth with respect to the control culture. Unsubstituted chalcone and safinamide were reported as reference.

**Table 1:**
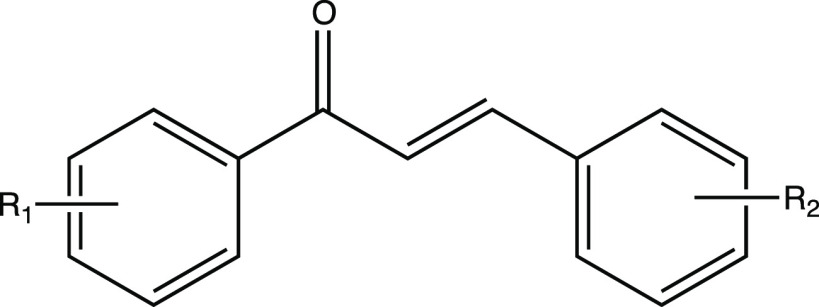

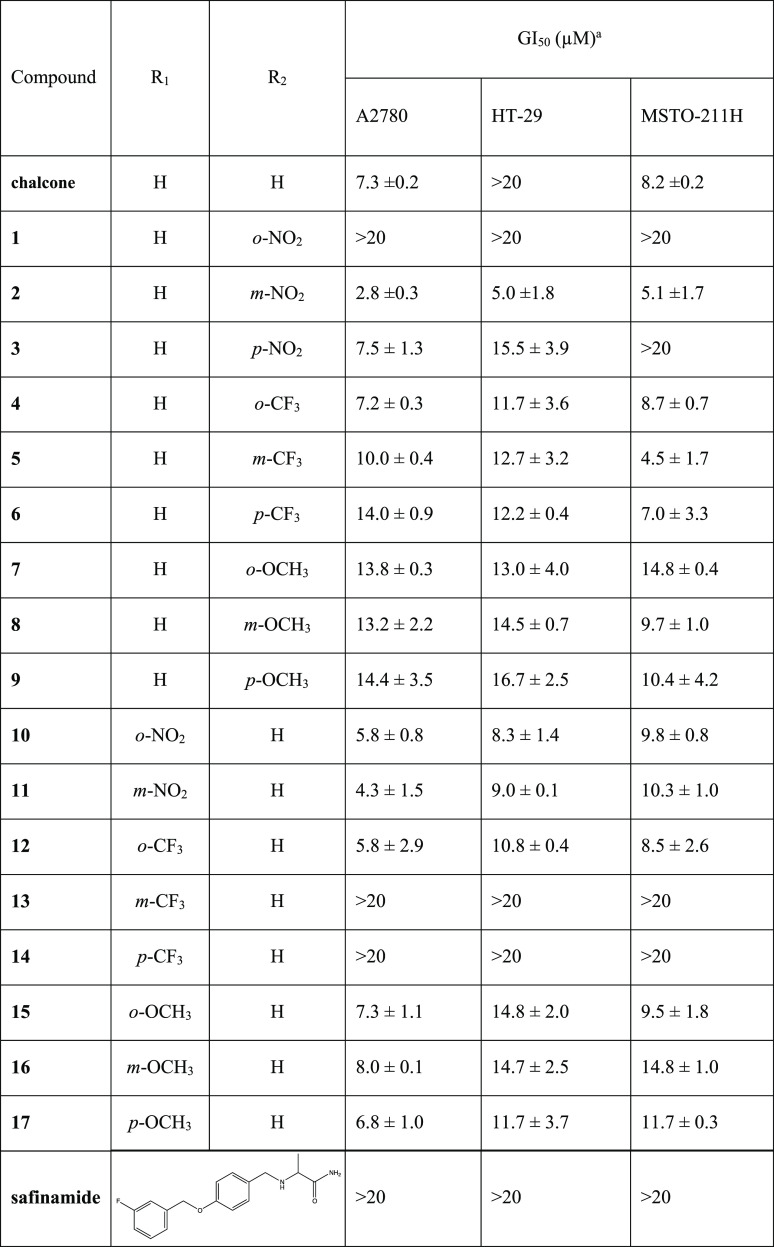
Cell Growth Inhibition after 72 h of Incubation in the Presence of the Examined Chalcones and Safinamide

a Values are reported as mean ± SD of at least three independent experiments in duplicate.

Interestingly, these results show that derivatives **1**, **13**, and **14** were unable to induce any cytotoxicity in any of the tested cell lines. Otherwise, the majority of the chalcones belonging to the selected library, as for the unsubstituted chalcone scaffold, induced a significant antiproliferative effect, with GI_50_ values ranging from 2.8 to 16.7 μM. In particular, in the series **1**–**9** bearing an electron-donating or electron-withdrawing R_2_ substituent, derivative **2**, characterized by the *m*-NO_2_ group, showed the highest cytotoxic effect in all of the considered cell lines (GI_50_ values ranging from 2.8 to 5.1 μM), while the *o*-NO_2_ analogue **1** was completely ineffective on cells. For all of the other derivatives (**3**–**9**), an intermediate effect was observed.

With regard to **10**–**17**, interesting differences in antiproliferative activity were observed for the derivatives bearing CF_3_ as the R_1_ substituent (**12**–**14**). In detail, while the *o*-CF_3_-substituted chalcone **12** was able to induce significant cytotoxicity, the insertion of the same group at the *meta* (**13**) or *para* (**14**) position abolished the effect on cells. For **10**–**11** and **15**–**17**, the position of the NO_2_ or OCH_3_ substituent, respectively, did not significantly affect the cytotoxicity. Finally, as expected, safinamide was completely ineffective on human cancer cells.

On the basis of the antiproliferative activity data (Table 1), the compounds devoid of detectable cytotoxic activity (GI_50_ > 20 μM) on all of the considered cell lines, *i.e.*, **1**, **13**, and **14**, were selected and evaluated as potential inhibitors for human recombinant MAO-A and -B enzymes using kynuramine (kyn) as the substrate. Moreover, the most cytotoxic derivative **2**, unsubstituted chalcone, and compound **17**, which had been previously studied,^19,23^ were also tested as reference compounds. Additionally, safinamide^24^ and isatin^25^ were included in the study as reversible standard inhibitors of MAOs.

All of the tested chalcones were found to inhibit both MAO-A and MAO-B. In particular, the Lineweaver–Burk double-reciprocal plots (1/*v*_0_ vs 1/[S]) of the kinetic data for MAO-B in the presence of various concentrations (5–40 nM) of **13** (Figure 1A) clearly demonstrate a competitive mode of inhibition, as only the *x*-intercept (−1/*K*_m_) is affected by the presence of the compound, in contrast to the *y*-intercept (1/*V*_max_), which is not significantly different at all tested concentrations. The reversibility of the inhibition was evaluated by incubating MAOs with **13** (at a concentration equal to 4 times the *K*_i_ value) for 20 min before starting dialysis cycles. After dialysis, the MAO activity was fully recovered, supporting the reversibility of the mode of inhibition of this compound. The same behavior was found for **1** and **14**, as can be clearly seen in Figure 1B, where results for the lead compound (chalcone) and safinamide are also shown. This competitive behavior has been reported previously for other chalcone derivatives.^20,23^

**Figure 1:**
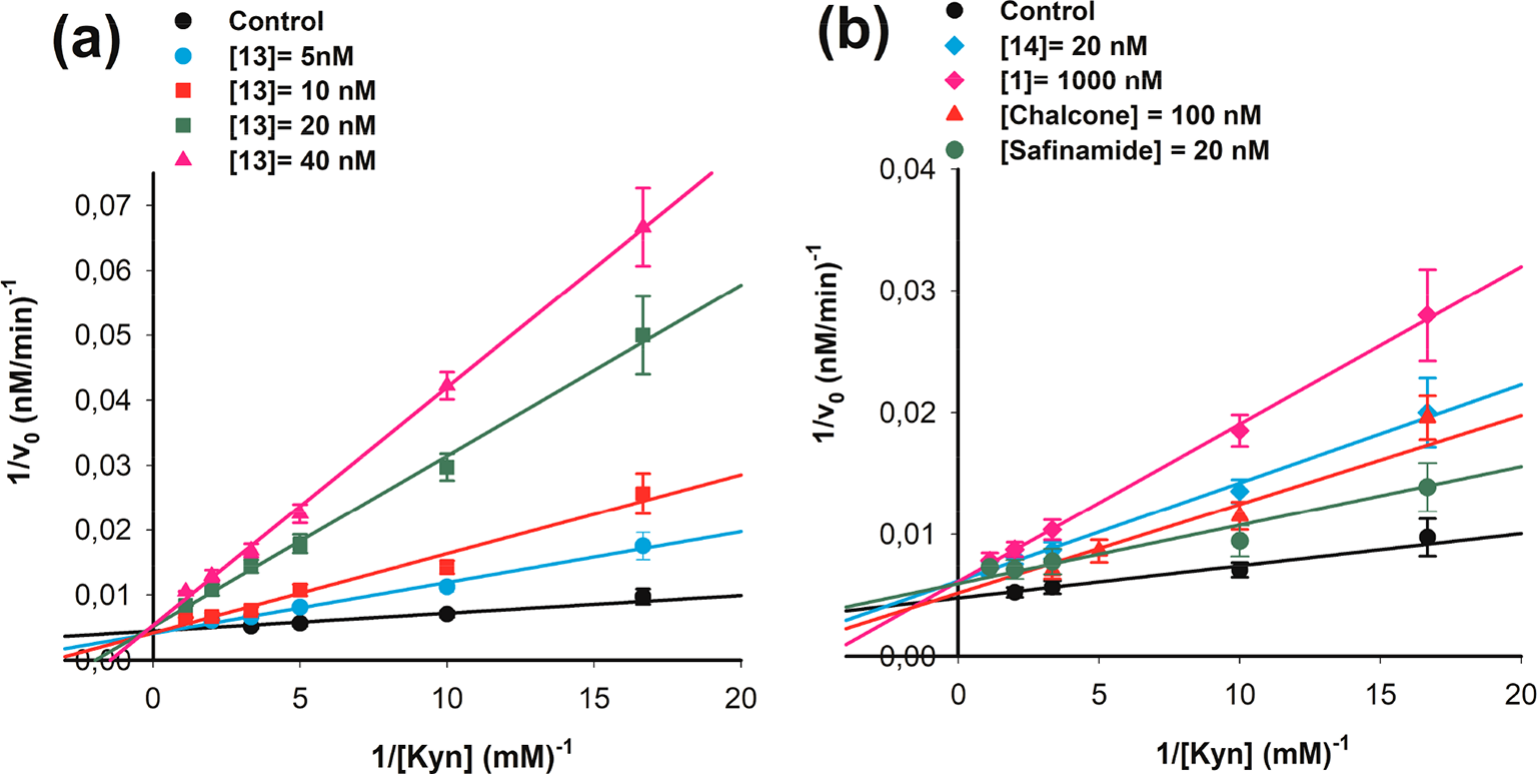
Competitive inhibition of chalcone derivatives on human MAO-B activity. Lineweaver–Burk double-reciprocal plots of MAO-B initial velocity vs substrate concentration (1/*v*_0_ vs 1/[kynuramine]) in the absence and presence of various concentrations of compounds. Continuous lines are the results of linear regression analysis of plotted data (*r* > 0.98). (a) Reciprocal plots at different concentrations of **13** (5–40 nM) showing that increasing the concentration of the compound increases the effect on *K*_m_ (from the *x*-intercept), while no effect on *V*_max_ (from the *y*-intercept) is observed. (b) Examples of reciprocal plots in the presence of **1**, **14**, and chalcone (the lead compound) that demonstrate their competitive behavior (effect on *K*_m_ only). Safinamide is shown as a standard competitive inhibitor. As these compounds have different inhibitory potencies, different concentrations were used to show the effect on MAO-B.

The values of the inhibition constant (*K*_i_) for **1**, **2**, **13**, **14**, and **17** were determined for both MAO-A and MAO-B and are reported in Table 2 in a comparative analysis with the standard inhibitors safinamide and isatin. From the data in Table 2, it clearly appears that the presence of the −CF_3_ group as the R_1_ substituent (at the *meta* (**13**) or *para* (**14**) position of the phenyl ring) significantly improves both the potency of the inhibition and the selectivity toward the MAO-B isoform with respect to unsubstituted chalcone. Otherwise, the insertion of the −NO_2_ group as the R_2_ substituent at the *meta* position (**1**) is detrimental to both MAO-B inhibition and the selectivity index (S.I.).

**Table 2:** Inhibition Constant Values and Selectivity Indices for Human Recombinant MAO-A and MAO-B by **1**, **13**, and **14** and Also by Chalcone, **2**, and **17** as Reference Chalcone Compounds and Safinamide and Isatin as Standard MAO Inhibitors

	*K*_i_ (nM)^a^		
compound	MAO-A	MAO-B	S.I.^b^
chalcone	(14.6 ± 1.0) × 10^3^	56 ± 6	260:1
**1**	(2.2 ± 0.2) × 10^3^	400 ± 80	5.5:1
**2**	(2.5 ± 1.0) × 10^3^	71 ± 11	35:1
**13**	(4.6 ± 0.5) × 10^3^	5.0 ± 0.5	920:1
**14**	(9.2 ± 1.8) × 10^3^	14.6 ± 0.1	630:1
**17**^c^	(13.0 ± 2.1) × 10^3^	21.9 ± 2.3	594:1
safinamide^d^	(82 ± 8) × 10^3^	17 ± 4	4820:1
isatin^e^	(16 ± 3) × 10^3^	(4 ± 1) × 10^3^	4:1

a Values are reported as mean ± SD of three independent experiments.

b Selectivity index, given by S.I. = *K*_i_(MAO-A)/*K*_i_(MAO-B).

c See refs (19) and (23).

d Standard MAO-B inhibitor.

e Standard MAO-A and MAO-B inhibitor.

In particular, compound **13** emerges as the most potent and selective inhibitor for MAO-B. Indeed, its inhibition constant value (*K*_i_ = 5.0 nM) is lower than that obtained for safinamide (*K*_i_ = 17 nM), and it shows very good selectivity (S.I. = 920 vs 4820 of safinamide). Finally, derivative **17** is less potent (*K*_i_ = 22 nM) and less selective (S.I. = 594) than **13** under our experimental conditions. It is worth mentioning that the *K*_i_ value reported in Table 2 for **17** is lower than that previously reported in the literature^19,23^ because it was determined under different experimental buffer and temperature conditions. In this regard, the **15**–**17** subseries was also tested previously on MAO-B, and for both **15** and **16** a lower inhibitory potency than for **17** was found.^19,23^

The noteworthy inhibitory potency (*K*_i_ value in the nanomolar range) and the high selectivity exerted by **13** point to this derivative as one of the most promising chalcone scaffolds for the development of agents for neurodegenerative disease. Indeed, comparable effects on MAO-B have been demonstrated only in a few trisubstituted^18^ and disubstituted^13^ chalcone derivatives and in a furan-based chalcone containing a cinnamyl group.^17^ The relevance of **13** is further supported by the absence of cytotoxicity also on nontumorigenic Met-5A mesothelial cells, for which a GI_50_ value higher than 20 μM was obtained, as for safinamide. In contrast, for the chalcone scaffold and **17**, cytotoxicity toward Met-5A was also confirmed, with GI_50_ values of about 7.0 ± 1.5 and 11.8 ± 1.1 μM, respectively.

Overall these results prompted us to perform molecular modeling studies on the most active compound of the series, *i.e.*, **13**.

First, *in silico* ADME predictions made with QikProp predicted that **13** has favorable drug-like properties and good blood–brain barrier permeability (see Table S1). Then, docking calculations into representative conformations of the human MAO-A and MAO-B proteins were performed (see the Supporting Information for the experimental details).^24,26^ In particular, two different binding modes between **13** and MAO-B were predicted (termed binding modes *a* and *b*), while a single binding mode was observed for MAO-A. Figure 2a shows the binding mode of **13** in MAO-A, and Figure 2b depicts the more stable binding mode of **13** in MAO-B (herein termed binding mode *b*). The less stable binding mode of **13** in MAO-B (binding mode *a*) is shown in Figure S1. The two binding modes in MAO-B are likely due to the peculiar morphology of this isoform, which is composed of two similar subcavities separated by the Ile199 and Tyr326 side chains.^27,28^ The binding modes are similar to those previously reported for other chalcone inhibitors.^21,29^

**Figure 2:**
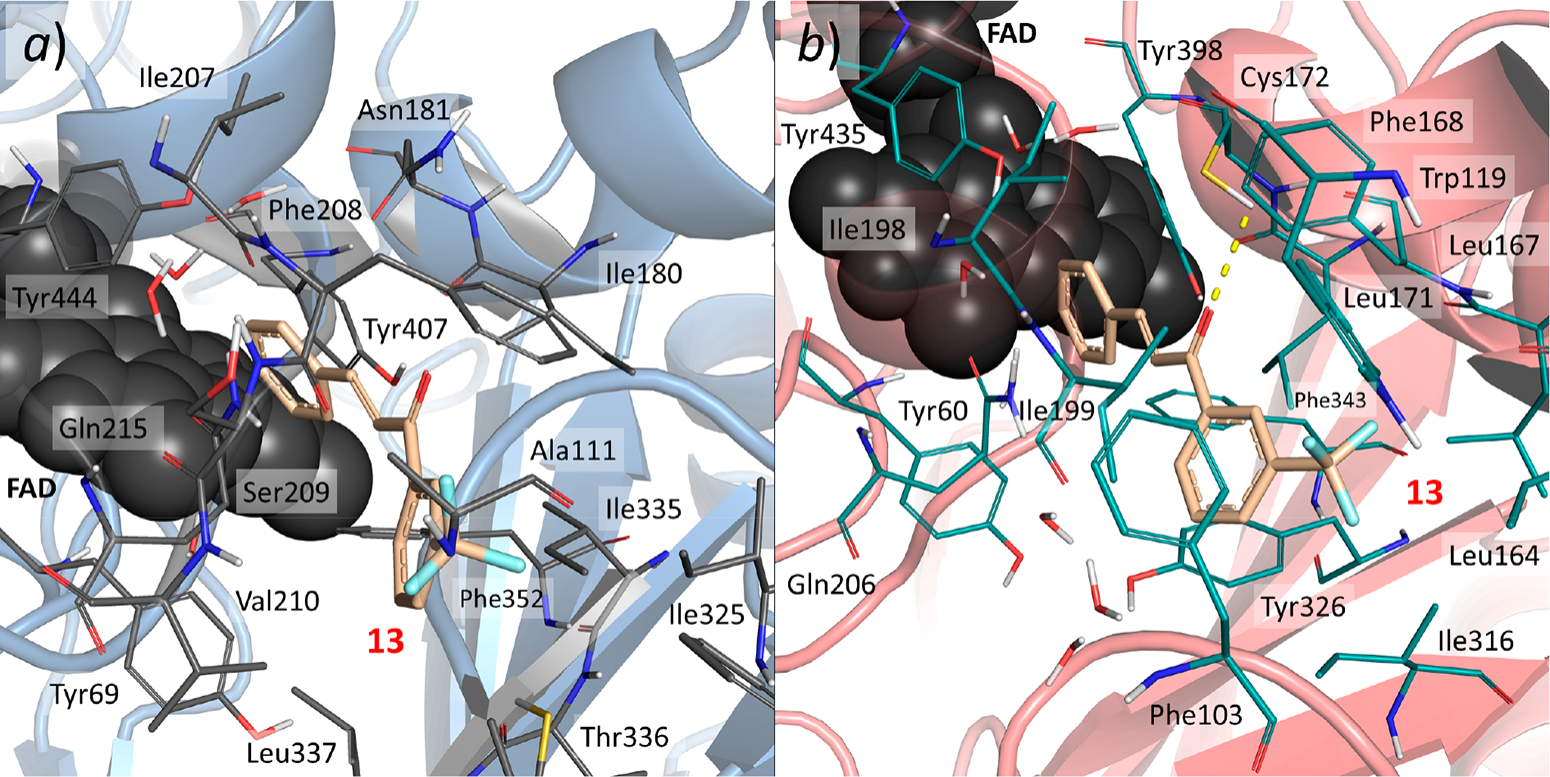
Predicted docking poses of **13** in the MAO-A and MAO-B crystal structures: (a) binding mode of **13** in the MAO-A protein (PDB ID 2Z5X); (b) the more energetically favored binding mode (binding mode *b*) predicted for **13** in the MAO-B crystal structure (PDB ID 2V5Z).

The docking scores in MAO-B were −10 kcal·mol^–1^ for binding mode *a* and −11 kcal·mol^–1^ for binding mode *b*. The docking score in MAO-A was −7.5 kcal/mol. To better discriminate between the two binding modes in MAO-B, a rescoring of the predicted docking poses was performed with a more rigorous free-energy-based screening methodology (*i.e.*, BEAR) to evaluate the binding free energy of the ligand.^30^

According to the binding free energy scores (Δ*G*_bind_) predicted by BEAR, binding mode *b* of **13** turned out to be 6.8 kcal·mol^–1^ more stable than binding mode *a* (Table S2). In binding mode *b*, the B phenyl ring of **13** establishes hydrophobic interactions with the aromatic rings of the Tyr398, Tyr435, and Tyr60 side chains and the flavine ring of FAD. The α,β-unsaturated ketone binds close to the Ile198 and Leu171 side chains, with the carbonyl moiety forming a hydrogen bond with the Cys172 side chain, as previously observed for other chalcone compounds.^29^ The A ring forms hydrophobic contacts with the Leu164, Leu167, Phe168 and Trp119 side chains, as previously observed in ref (29). In particular, the trifluoromethyl moiety was predicted to be accommodated near the Trp119 and Leu164 side chains, establishing close contacts. The hydrogen bond of the carbonyl with the Cys172 residue is of special interest because this residue, which is mutated to Asn181 in MAO-A, has a recognized role in MAO-B/MAO-A selectivity.^21,29^ Importantly, docking of **13** into MAO-A did not provide an orientation similar to that of MAO-B. In MAO-A, the trifluoromethylbenzene moiety (ring A) was predicted to be accommodated toward the entrance of the MAO-A binding site near residues Leu97, Ile335, and Leu337 because of steric clashes with the Phe208 side chain. Moreover, the carbonyl group of **13** did not establish hydrogen bonds with MAO-A residues. Interestingly, the lack of a hydrogen bond with residues in this region of the MAO-A binding pocket, like those previously observed with Cys172 or Tyr326 into MAO-B, has been reported to play an important role in isoform selectivity for the MAO proteins.^27,28,31^ The B phenyl ring is accommodated close to the Tyr60 and Tyr407 side chains and FAD, similar to what was observed in MAO-B. Altogether, these differences help explain the experimentally observed selectivity of compound **13** for MAO-B over MAO-A.

Considering that docking calculations on **13** suggested two alternative orientations, one of which was clearly favored over the other, we sought to investigate whether *para* (compound **14**) instead of *meta* (compound **13**) trifluorocarbon substitution could play a role in discriminating the ligand orientation. To this aim, the binding modes of **13** and **14** in MAO-B were studied by X-ray crystallography. The three-dimensional structures of human MAO-B in complex with **13** (Figures 3 and 4a) and **14** (Figure 4b) were solved at 2.3 and 2.1 Å resolution, respectively (statistics are reported in Table S3). For both structures, the overall fold and, in particular, the active site and the bound inhibitor display the same conformation between the two monomers of the dimer contained in the asymmetric unit (the root-mean-square deviations for Cα atoms are 0.30 and 0.27 Å between chains A and B for the structures in complex with compounds **13** and **14**, respectively). Hereafter, we will refer to subunit A of each structure for the following discussion.

**Figure 3:**
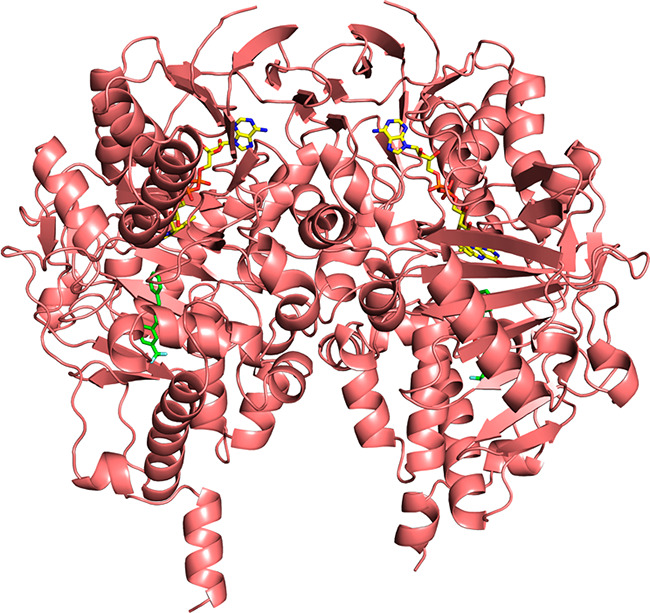
Overall structure of the MAO-B dimer represented as a pink ribbon diagram (chain A is on the left) with the membrane-spanning C-terminal helix pointing to the bottom of the figure. The FAD cofactor is shown in stick representation with carbon, nitrogen, oxygen, and phosphorus atoms colored in yellow, blue, red, and magenta, respectively. The structure in complex with **13** (in green, with fluorine atoms in light blue and oxygen in red) is shown. The overall fold is identical to that of the enzyme in complex with **14**.

**Figure 4:**
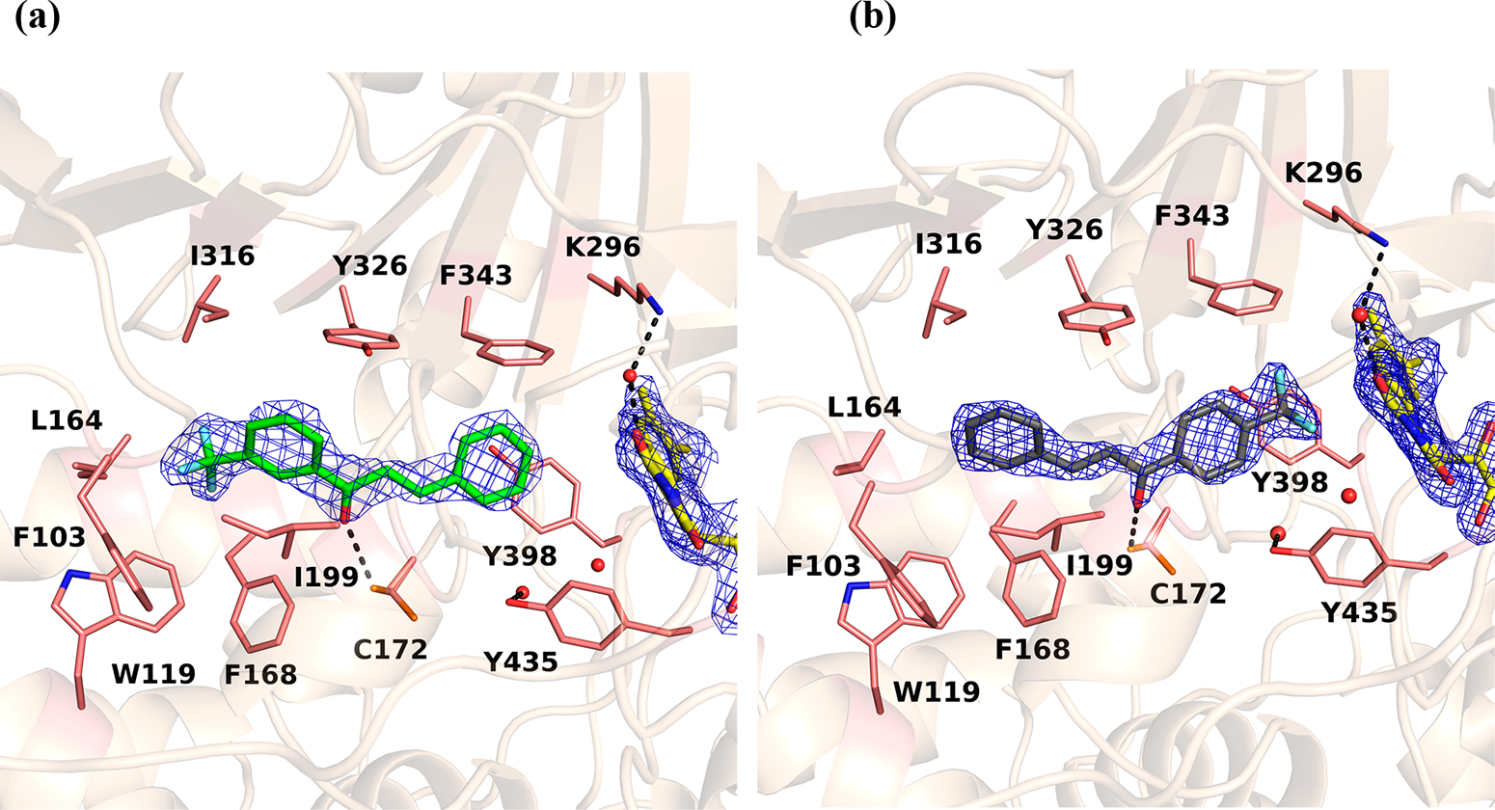
Architectures of the MAO-B active site in complex with (a) **13** and (b) **14**. FAD is depicted as in Figure 3. The residues that line the cavity are represented as sticks with carbon, nitrogen, oxygen, and sulfur atoms in pink, blue, red, and orange, respectively. The refined 2*F*_o_ – *F*_c_ electron density map for the inhibitor molecule (contoured at 1.2σ) is shown in blue chicken-wire style. The fluorine atoms of both inhibitors are in light blue, with carbon atoms in green (**13**) and gray (**14**). Water molecules are depicted as red spheres and hydrogen bonds as dashed lines.

Inspection of the electron density in the enzyme active site revealed that **13** and **14** bind in the hydrophobic cavity of MAO-B (Figure 4a,b, respectively). In both cases the chalcone moiety is accommodated with the carbonyl oxygen pointing toward the bottom of the active site and establishing a hydrogen bond with Cys172 in one of the two conformations that this residue adopts. This feature was also found in the MAO-B structures in complex with chromone inhibitors.^32^ The distances between the cysteine side chain and the chalcone alkene unit are 4.0 and 4.6 Å for **13** and **14**, respectively, both of which are too long for the formation of a covalent bond as previously observed for other targets.^33^ Compound **13** binds with the CF_3_ substituent in the entrance cavity space (Figure 4a), which is in agreement with the most stable orientation predicted by modeling as described above and is consistent with previous studies highlighting this part of the active site as a favorable niche for halogen atoms (such as in the case of safinamide; see Figure 5). Most likely, in the crystallized MAO-B–**13** complex the predicted binding mode *b* of **13** is selected. Interestingly, **14** is bound in the opposite way with respect to the flavin compared with **13**, with the CF_3_ group positioned within the aromatic cage formed by residues Tyr398 and Tyr435 in front of the flavin (Figure 4b). This is unique in MAO-B structures and is probably due to the *para* position of the substituent on the aromatic ring, which would clash with the residues belonging to the gating loop formed by residues 99–110 (on the left in Figure 4). Similar to the fluorine atom substituent in safinamide, the CF_3_ group of **13** is at the *meta* position and can easily be accommodated in the entrance cavity. Superposition of the MAO-B structures in complex with the two chalcone inhibitors highlights that the carbonyl–Cys172 hydrogen bond functions as a sort of pivot point by fixing the molecules in the active site and orienting them in opposite directions with respect to the flavin (Figure 5). The chalcone scaffold is constrained by the flat and rigid MAO-B cavity to position the inner aromatic ring perpendicular to the flavin and the other slightly tilted, similar to the conformation observed in safinamide (Figure 5). Retrospective docking calculations predicted only one binding mode for **14** (docking score of −10 kcal·mol^–1^), consistent with the orientation observed in the crystal structure and similar to the predicted binding mode *a* of **13** (Figure S2).

**Figure 5:**
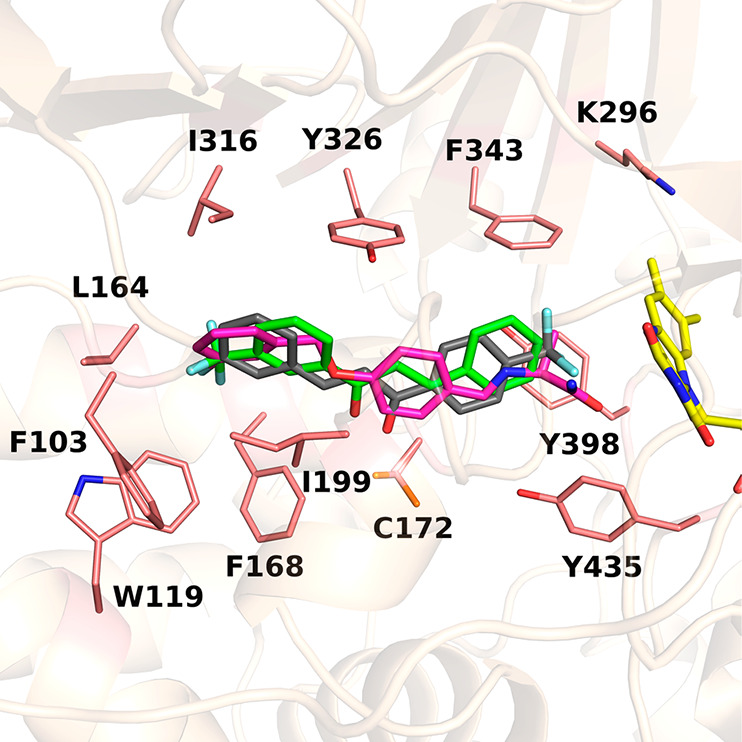
Superposition of MAO-B structures in complex with **13**, **14**, and safinamide (the latter represented with carbon atoms in magenta; PDB code 2V5Z).^24^ For the sake of clarity, water molecules and hydrogen bonds shown in Figure 4 have been omitted.

In conclusion, we report that among a library of monosubstituted structurally related chalcones, three derivatives (**1**, **13**, and **14**) demonstrated a safe profile on different human cell lines and then were considered for further investigation. In particular, **13** appeared to be good candidate to target MAO enzymes in the context of neurodegenerative diseases. Indeed, it behaves as competitive reversible inhibitor of MAO-B, showing an inhibitory potency of *K*_i_ = 5 nM (which is higher than that of the clinically used safinamide) and a remarkable selectivity toward the MAO-B isoform. Docking studies of the binding of **13** to MAOs confirmed the strong affinity and selectivity for MAO-B versus MAO-A by predicting opposite orientations in the active site of the two isoforms (with the trifluoromethylbenzene moiety pointing toward the FAD cofactor in MAO-B and toward the active-site entrance in MAO-A). Crystal structures of MAO-B in complex with **13** and **14** highlighted the key role played by the position of the trifluoromethyl moiety on the phenyl ring (*meta* in the former and *para* in the latter) in orienting the inhibitor into the active site.

Overall, our studies pointed to **13** as a very promising chalcone derivative to be considered for the development of novel MAO-B-selective inhibitors for the treatment of neurodegenerative diseases. The evaluation of its effectiveness and nontoxicity *in vivo* and the effect on other targets involved in neurodegenerative disorders warrant further investigation.

## Supporting Information Available

Chemistry, materials and methods, molecular docking, crystallographic details, and NMR spectra (PDF)
