# Supplementary material for: Promising Non-cytotoxic Monosubstituted Chalcones to Target Monoamine Oxidase-B
Source: ACS Med Chem Lett. 2021 Jun 14;12(7):1151–8. doi: 10.1021/acsmedchemlett.1c00238 (PMC8274062; doi:10.1021/acsmedchemlett.1c00238)
Supplement: Supplementary file 1 — ml1c00238_si_001.pdf [file ml1c00238_si_001.pdf]

Supporting Information

Promising non-cytotoxic monosubstituted chalcones to target monoamine oxidase-B.

Luca G. Iacovino,[a,#] Luca Pinzi,[b,#] Giorgio Facchetti,[c,#] Beatrice Bortolini,[d] Michael S. Christodoulou,[c,]* Claudia Binda,[a] Giulio Rastelli,[b] Isabella Rimoldi,[c] Daniele Passarella,[e] Maria Luisa Di Paolo,[f] Lisa Dalla Via[d,]*

[#] These authors contributed equally

[a]*Dipartimento di Biologia e Biotecnologie, Università di Pavia, via Ferrata 9, 27100, Pavia, Italy*
[b]*Dipartimento di Scienze della Vita, Università degli Studi di Modena e Reggio Emilia, via Campi, 103, 41125, Modena, Italy*
[c]*DISFARM, Sezione di Chimica Generale e Organica "A. Marchesini" Università degli Studi di Milano via Venezian, 21, 20133, Milano, Italy*
[d]*Dipartimento di Scienze del Farmaco, Università degli Studi di Padova, via F. Marzolo, 5, 35131, Padova, Italy*
[e]*Dipartimento di Chimica, Università degli Studi di Milano, via C. Golgi, 19, 20133, Milano, Italy*
[f]*Dipartimento di Medicina Molecolare, Università degli Studi di Padova, via G. Colombo, 3, 35131, Padova, Italy*

* Corresponding author: lisa.dallavia@unipd.it; michail.christodoulou@unimi.it

Chemistry	S3
Materials and Methods	S6
Table S1	S14
Table S2	S15
Table S3	S16
Figure S1	S17
Figure S2	S18
NMR spectra	S19

CHEMISTRY

General. ^{1}H and ^{13}C NMR spectra were recorded in $CDCl_3$ on Bruker DRX Avance (300 and 75 MHz) equipped with a non-reverse probe. Chemical shifts (in ppm) were referenced to residual solvent proton/carbon peak.

Synthesis of α,β-Unsaturated ketones

An aqueous solution of sodium hydroxide (30% *p/p*, 20 mL) was slowly added to a methanolic solution of the chosen acetophenone (1 eq in 20 mL) at 0°C. The solution was cooled to room temperature and the appropriate benzaldehyde (1.2 eq.) was added. The mixture was stirred at room temperature overnight and was then poured into water (50 mL). The obtained solid was filtered, washed with water until neutral pH and recrystallized twice from ethanol.

(E)-3-(2-nitrophenyl)-1-phenylprop-2-en-1-one **1***:* ^{1}H NMR (300 MHz, $CDCl_3$) δ 8.24-8.15 (m, 4H), 7.89-7.82 (m, 1H), 7.76-7.71 (m, 1H), 7.57-7.45 (m, 4H) ppm. ^{13}C NMR (75 MHz, $CDCl_3$) δ 157.36, 148.32, 139.71, 136.73, 129.77, 129.62, 129.30, 128.82, 127.57, 127.44, 127.19, 126.26, 118.98 ppm.

(E)-3-(3-nitrophenyl)-1-phenylprop-2-en-1-one **2**: ^{1}H NMR (300 MHz, $CDCl_3$) δ 8.49 (s, 1H), 8.23 (d, *J*=8.9 Hz, 1H), 8.03 (d, *J*=8.7 Hz, 1H), 7.91 (d, *J*=6.5 Hz, 1H), 7.82 (d, *J*=12.4 Hz, 1H), 7.67-7.49 (m, 5H) ppm. ^{13}C NMR (75 MHz, $CDCl_3$) δ 189.60, 147.12, 141.57, 137.58, 136.66, 134.26, 133.27, 130.02, 128.78, 128.58, 124.64, 124.62, 122.35 ppm.

(E)-3-(4-nitrophenyl)-1-phenylprop-2-en-1-one **3**: ^{1}H NMR (300 MHz, $CDCl_3$) δ 8.29 (d, *J*=8.9 Hz, 2H), 8.07 (d, *J*=8.8 Hz, 2H), 7.82-7.79 (m, 3H), 7.69-7.28 (m, 4H) ppm. ^{13}C NMR (75 MHz, $CDCl_3$) δ 190.02, 148.94, 141.90, 141.44, 137.92, 133.78, 129.34, 129.22, 128.99, 126.10, 124.61 ppm.

(E)-1-phenyl-3-(2-(trifluoromethyl)phenyl)prop-2-en-1-one **4:** ^{1}H NMR (300 MHz, $CDCl_3$) δ 8.12 (dd, *J*=8.9 Hz, *J*=3.0 Hz, 1H) , 8.03-8.00 (m, 2H), 7.83 (d, *J*=11.8 Hz, 1H), 7.73 (d, *J*=12.1 Hz, 1H), 7.64-7.57 (m, 2H), 7.54-7.48 (m, 3H), 7.43 (d, *J*=14.3 Hz, 1H) ppm. ^{13}C NMR (75 MHz, $CDCl_3$) δ 190.31, 140.23, 137.67, 134.06, 133.01, 132.08, 129.84, 129.64, 129.64, 129.03, 128.66, 127.95, 126.64, 126.37, 126.29, 126.22, 126.14, 125.76, 122.13, 118.50 ppm.

(E)-1-phenyl-3-(3-(trifluoromethyl)phenyl)prop-2-en-1-one **5**: ^{1}H NMR (300 MHz, $CDCl_3$) δ 8.05 (d, *J*=11.3 Hz, 2H), 7.89-7.79 (m, 3H), 7.68-7.49 (m, 6H) ppm. ^{13}C NMR (75 MHz, $CDCl_3$) δ 189.98, 142.76, 137.84, 135.74, 133.05, 132.19, 131.76, 131.56, 131.54, 131.33, 130.37, 129.50, 128.71, 128.69, 128.60, 128.54, 126.85, 126.80, 126.75, 126.70, 125.61, 124.75, 124.70, 124.65, 124.59, 123.75, 121.99, 119.05 ppm.

(E)-1-phenyl-3-(4-(trifluoromethyl)phenyl)prop-2-en-1-one **6**: ^{1}H NMR (300 MHz, $CDCl_3$) δ 8.04 (d, *J*=8.5 Hz, 2H), 7.84-7.50 (m, 9H) ppm. ^{13}C NMR (75 MHz, $CDCl_3$) δ 189.52, 146.86, 142.64, 138.33, 137.84, 133.06, 132.01, 131.13, 130.94, 128.70, 128.52, 128.45, 127.85, 126.80, 125.91, 125.91, 125.86, 124.37 ppm.

(E)-3-(2-methoxyphenyl)-1-phenylprop-2-en-1-one **7**: ^{1}H NMR (300 MHz, $CDCl_3$) δ 8.13 (d, *J*=15.6 Hz, 1H), 8.04 (d, *J*=7.4 Hz, 2H), 7.65-7.42 (m, 5H), 7.35 (t, *J*=4.3 Hz, 1H), 7.02-6.93 (m, 2H), 3.91 (s, 3H) ppm. ^{13}C NMR (75 MHz, $CDCl_3$) δ 191.11, 158.83, 140.37, 138.57, 132.48, 131.71, 129.22, 128.51, 124.00, 122.96, 120.75, 111.28, 55.54 ppm.

(E)-3-(3-methoxyphenyl)-1-phenylprop-2-en-1-one **8**: ^{1}H NMR (300 MHz, $CDCl_3$) δ 8.02 dd, *J*=1.5 Hz, 11.5 Hz, 2H), 7.77 (d, *J*=14.8 Hz, 1H), 7.61-7.47 (m, 4H), 7.35-7.15 (m, 3H), 6.98-6.94 (m, 1H), 3.84 (s, 3H) ppm. ^{13}C NMR (75 MHz, $CDCl_3$) δ 190.50, 159.98, 144.72, 138.21, 136.28, 132.77, 129.94, 128.62, 128.54, 128.51, 122.43, 121.09, 116.31, 113.48, 55.33 ppm.

(E)-3-(4-methoxyphenyl)-1-phenylprop-2-en-1-one **9**: ^{1}H NMR (300 MHz, $CDCl_3$) δ 8.04 (d, *J*=8.1 Hz, 2H), 7.77 (d, *J*=13.7 Hz, 1H), 7.62-7.41 (m, 6H), 6.87 (d, *J*=7.8 Hz, 2H), 3.85 (s, 3H) ppm. ^{13}C NMR (75 MHz, $CDCl_3$) δ 190.58, 161.70, 144.67, 138.55, 132.51, 130.20, 129.05, 128.54, 128.40, 128.02, 127.66, 119.86, 114.43, 113.62, 55.13 ppm.

(E)-1-(2-nitrophenyl)-3-phenylprop-2-en-1-one **10**: ^{1}H NMR (300 MHz, $CDCl_3$) δ 8.02 (d, *J*=7.8 Hz, 1H), 7.77 (t, *J*=3.8 Hz, 1H), 7.62 (d, *J*=3.3 Hz, 1H), 7.52-7.47 (m, 3H), 7.44-7.39 (m, 3H), 7.24 (d, *J*=14.2 Hz, 1H), 7.0 (d, *J*=14.8 Hz, 1H) ppm. ^{13}C NMR (75 MHz, $CDCl_3$) δ 192.85, 146.80, 146.27, 136.32, 134.00, 133.97, 131.04, 130.58, 128.99, 128.81, 128.69, 128.54, 126.26, 124.52 ppm.

(E)-1-(3-nitrophenyl)-3-phenylprop-2-en-1-one **11**: ^{1}H NMR (300 MHz, $CDCl_3$) δ 8.84 (s, 1H), 8.40 (dd, *J*=3.3 Hz, *J*=17.8 Hz, 2H), 7.92 (d, *J*=15.3 Hz, 1H), 7.74-7.66 (m, 3H), 7.54-7.41 (m, 4H) ppm. ^{13}C NMR (75 MHz, $CDCl_3$) δ 187.99, 148.44, 146.75, 139.52, 134.33, 134.06, 131.19, 129.89, 129.09, 128.72, 127.03, 123.25, 120.69 ppm.

(E)-3-phenyl-1-(2-(trifluoromethyl)phenyl)prop-2-en-1-one **12**: ^{1}H NMR (300 MHz, $CDCl_3$) δ 7.79-7.73 (m, 1H), 7.67-7.59 (m, 2H), 7.58-7.33 (m, 6H), 7.29 (d, *J*=14.9 Hz, 1H), 7.07 (d, *J*=15.3 Hz, 1H) ppm. ^{13}C NMR (75 MHz, $CDCl_3$) δ 194.97, 147.67, 138.93, 134.14, 131.61, 131.01, 130.45, 129.82, 129.00, 128.53, 128.36, 128.08, 127.68, 126.83, 126.81, 126.72, 126.66, 126.59, 125.44, 121.81 ppm.

(E)-3-phenyl-1-(3-(trifluoromethyl)phenyl)prop-2-en-1-one **13**: ^{1}H NMR (300 MHz, $CDCl_3$) δ 8.27 (s, 1H), 8.23 (d, *J*=7.8 Hz, 1H), 7.89-7.83 (m, 2H), 7.69-7.65 (m, 3H), 7.52 (d, *J*=14.8 Hz, 1H), 7.46-7.43 (m, 3H) ppm. ^{13}C NMR (75 MHz, $CDCl_3$) δ 188.98, 146.00, 138.80, 134.55, 131.56, 131.04, 130.90, 129.25, 129.10, 129.06, 129.01, 128.58, 125.57, 125.27, 125.22, 125.17, 121.96, 121.25 ppm.

(E)-3-phenyl-1-(4-(trifluoromethyl)phenyl)prop-2-en-1-one **14**: ^{1}H NMR (300 MHz, $CDCl_3$) δ 8.11 (d, *J*=8.7 Hz, 2H), 7.85 (d, *J*=14.7 Hz, 1H), 7.76 (d, *J*=9.3 Hz, 2H), 7.68-7.65 (m, 2H), 7.52 (d, *J*=15.4 Hz, 1H), 7.46-7.44 (m, 3H) ppm. ^{13}C NMR (75 MHz, $CDCl_3$) δ 189.96, 146.46, 141.49, 141.47, 141.46, 141.44, 135.04, 134.93, 134.61, 134.18, 133.74, 131.36, 129.53, 129.45, 129.17, 129.01, 126.12, 126.07, 126.02, 125.97, 125.91, 122.31, 121.96, 118.68 ppm.

(E)-1-(2-methoxyphenyl)-3-phenylprop-2-en-1-one **15**: ^{1}H NMR (300 MHz, $cdcl_3$) δ 7.66-7.61 (m, 2H), 7.57-7.54 (m, 2H), 7.47-7.34 (m, 5H), 7.04-6.95 (m, 2H), 3.84 (s, 3H) ppm. ^{13}C NMR

(75 MHz, cdcl$_3$) δ 192.77, 158.17, 143.07, 135.15, 132.94, 130.32, 130.25, 129.30, 128.90, 128.40, 128.38, 127.15, 120.75, 111.77, 55.74 ppm.

(E)-1-(3-methoxyphenyl)-3-phenylprop-2-en-1-one **16**: ^{1}H NMR (300 MHz, CDCl$_3$) δ 7.82 (d, *J*=14.9 Hz, 1H), 7.66-7.56 (m, 2H), 7.54 (d, *J*=15.1 Hz, 1H), 7.49-7.38 (m, 4H), 7.1 (dd, *J*=2.9 Hz, *J*=7.9 Hz, 1H), 3.89 (s, 3H) ppm. ^{13}C NMR (75 MHz, CDCl$_3$) δ 190.21, 159.95, 144.79, 139.65, 134.94, 130.49, 129.54, 128.93, 128.42, 122.23, 121.03, 119.24, 112.95, 55.46 ppm.

(E)-1-(4-nitrophenyl)-3-phenylprop-2-en-1-one **17**: ^{1}H NMR (300 MHz, CDCl$_3$) δ 8.05 (d, *J*=7.9 Hz, 2H), 7.80 (d, *J*=12.9 Hz, 1H), 7.66,-7.63 (m, 2H), 7.55 (d, *J*=13.8 Hz, 1H), 7.42-7.40 (m, 3H), 7.00, 6.99 (d, *J*=7.8 Hz, 2H), 3.88 (s, 3H) ppm. ^{13}C NMR (75 MHz, CDCl$_3$) δ 188.69, 163.44, 143.91, 135.12, 131.14, 130.79, 130.28, 128.90, 128.33, 121.96, 113.85, 55.46 ppm

MATERIALS AND METHODS

Materials. Human recombinant MAO-A and MAO-B were expressed in baculovirus infected BT1 cells (5 mg/ml) and horseradish peroxidases were purchased from Fluka-Sigma-Aldrich s.r.l. (Italy). A Cary-Eclipse fluorimeter (Varian Inc., Palo Alto, CA, USA) was employed for fluorescence measurements. The stock solutions of chalcone derivatives and safinamide were prepared in dimethylsulfoxide.

Cell growth assay. A2780 (human ovarian carcinoma cells) and HT29 (human colorectal adenocarcinoma) were grown in RPMI-1640 (Sigma Chemical Co.), MSTO-211H (human biphasic mesothelioma cells) and Met-5A (human mesothelial cells) were grown in RPMI-1640 (Sigma Chemical Co.) supplemented with 2.38 g/L Hepes, 0.11 g/L pyruvate sodium and 2.5 g/L glucose. 10% Heat-inactivated fetal calf serum (Biowest), 100 U/mL penicillin, 100 μg/mL streptomycin and 0.25 μg/mL amphotericin B (Sigma Chemical Co.) were added to the media. The cells were cultured at 37 °C in a moist atmosphere of 5% carbon dioxide in air. To determine the growth inhibition, cells ($3\text{-}4\times10^4$) were seeded into each well of a 24-well cell culture plate. After incubation for 24 h various concentrations of the test agents were added and the cells were then incubated in standard conditions for a further 72 h. A trypan blue assay was performed to determine cell viability. Cytotoxicity data were expressed as GI_{50} values, i.e. the concentration of the test agent inducing 50% reduction in cell number compared with control cultures.

Monoamine oxidase inhibition assay. Monoamine oxidase activity was determined by measuring H_2O_2 generation rate by a peroxidase-coupled continuous assay, using the Amplex Red reagent as fluorogenic substrate for horseradish peroxidase.[1] All experiments were carried out as previously reported,[2] in 100 mM potassium phosphate buffer, 0.1 mM EDTA, pH 7.4 and at 37°C. The assays were carried out in a final volume of 800 μL, in the presence of Amplex Red (100 μM) and horseradish peroxidase type II (5 UmL^{-1}), and using kynuramine (20-1200 μM) as common MAO-A and MAO-B substrate. h-MAO-A and h-MAO-B concentration in the assay solutions were 3 and 6 μg/ml, respectively. After 5 min of pre-incubation of the enzyme with the compounds, substrate was added and the initial rates of reaction were recorded. The rates of the enzymatic reaction were determined by detecting the increase in the fluorescence intensity at λ_{em}= 586 nm (λ_{exc}= 563nm). The generation rate of H_2O_2 was calculated from the increase of fluorescence intensity by means of calibration curves obtained using standard solution of H_2O, in absence of MAO enzymes. No interference of the chalcone derivatives on fluorescence intensity (calibration curves) was observed in the explored concentrations of the tested compounds (20 μM of maxima concentrations tested).

Steady state kinetic parameters (V_{max} and K_m) were calculated by fitting the Michaelis-Menten equation to the experimental data (initial velocity *vs* substrate concentrations), with Sigma Plot software, version 9.0 (Jandel Scientific, San Rafael, CA, USA). The mode of inhibition was determined by global fit analysis (GraphPad 9.0 software, GraphPad Software, San Diego, CA,

USA) of the initial velocity (V_0) *vs* substrate concentration plots, at various inhibitor concentrations. The competitive, mixed, non-competitive and uncompetitive inhibition models were analysed and the fit giving the highest r^2 value was chosen for the calculation of inhibition constants (K_i). For a better visualization of the competitive mode of inhibition of the tested compounds, experimental data are presented as double reciprocal Lineweaver-Burk plots (1/v *vs* 1/S). Ki values are expressed as mean ±S.D. All experiments were carried out at least in triplicate.

Reversibility assay. Reversibility of the inhibition of MAO was evaluated by incubating the enzyme (recombinant MAO-A or MAO-B, at 0.0012 μg/mL) with **13** (the most potent MAO-B inhibitor, see Table 2) at concentration equal to fourfold of its Ki values, for 20 min, at 37°C, in 10 mL potassium phosphate buffer (0.1 M K/Pi, pH 7.4, 0.1 mM EDTA). Then, the enzyme-inhibitor (or the control sample without inhibitor) solution was centrifuged and concentrated fifty-fold using a Vivaspin 6 -10000 MWCO PES- ultrafiltration concentrators (Sartorius Italy, s.r.l., Varedo MB, Italy) (3000 x g, 16 min at 15°C). After re-dilution with 10 mL of potassium phosphate buffer and centrifugation, in the recovered solution, the enzyme was retained by the membrane while the inhibitor was not and consequently MAOs were concentrated in comparison to the initial solution. These recovered solutions were diluted to assay the residual MAO activity, in saturating concentration of substrate ([Kyn]=500 μM). A control sample, in the absence of inhibitor, was run under the same experimental conditions.

Protein preparation for docking studies. X-ray crystal structures of human monoamine oxidases A and B (MAO) in complex with ligands were firstly collected from the PDB (accessed on September 20, 2019), and complexes were split in their component chains. Then, the resulting structures were pre-treated by using the Protein Preparation Wizard utility available within the Schrödinger suite (release 2018-3), with default parameters.[3,4] Missing amino acid side chains were rebuilt, and mismatches in the atom types and bond connectivity were fixed. Moreover, the complexes were optimized by using a series of constraints on the atom movements (root mean square - RMS threshold equal to 0.3Å). Afterward, co-crystallized ions, organic solvent molecules and waters were removed from the resulting structures, except for the 718, 725, 726 and 739 (MAO-A), and 1088, 1143, 1144, 1155, 1170, 1229, 1230 and 1351 (MAO-B) water molecules, which are highly conserved among the reported crystal structures.[5] Finally, the structures were aligned by using the Protein Structure Alignment utility available within the Schrödinger suite (release 2018-3) and ligands were extracted from the complexes in their crystallographic conformation.[6] This analysis allowed to build two datasets of ligands from 4 (MAO-A) and 41 (MAO-B) crystal structures. The Flavin-Adenine Dinucleotide (FAD) cofactor was considered as part of the protein.

Protein conformation selection. The selection of the most suitable protein conformations for docking was performed through the similarity with the co-crystallized ligands. In particular, 2D and 3D similarity assessments were performed by using the previously extracted MAO crystallographic ligands as reference queries. The chalcone inhibitor was prepared for the ligand-

based analyses, by firstly generating all the combinations of ionization states and tautomers potentially present at physiological pH with *LigPrep*.[7] Then, up to 600 conformers were generated for each of the pre-treated compounds with the OMEGA2 software (OpenEye) using default parameters.[8,9] ROCS (OpenEye) was used as an engine for the 3D similarity estimation, using default parameters.[10,11] The Tanimoto COMBO score (*i.e.*, the sum of the ShapeTanimoto and ColorTanimoto similarity indexes) was used to estimate the 3D similarity. 2D similarity analyses were performed by using three different types of molecular fingerprints, namely MACCS and ECFP4, ECFP6, implemented into the OpenEye python toolkits.[12] Results of the analyses (see Table A) indicated a low degree of similarity, but allowed the identification of PDB ID 2Z5X (chain A) and PDB ID 2V5Z (chain A) as the most suitable protein conformations for docking into MAO-A and MAO-B isoforms, respectively.[13,14] In particular, safinamide, which is in complex with MAO-B in the 2V5Z crystal structure, resulted to be the most similar ligand to 13 according to the ECFP4 and ECFP6 indexes (Table A).

Table A: Crystal structures used in the analyses. Structures selected for the docking calculations are highlighted in bold. Similarity score values above commonly accepted thresholds of similarity are highlighted in red.

MAO-B ligand	*Compound ID*	*MACCSfp score*	*ECFP4fp score*	*ECFP6fp score*	*Tanimoto Combo*	*Shape Tanimoto*	*Color Tanimoto*
2V5Z_chainB	13	0.314	0.433	0.302	0.791	0.543	0.248
**2V5Z_chainA**	**13**	**0.314**	**0.433**	**0.302**	**0.760**	**0.536**	**0.224**
2C70_chainA	13	0.135	0.375	0.256	0.822	0.489	0.334
2C70_chainB	13	0.135	0.375	0.256	0.822	0.488	0.334
1OJC_chainA	13	0.2	0.333	0.222	0.954	0.621	0.333
1OJC_chainB	13	0.2	0.333	0.222	0.953	0.621	0.333
2VRM_chainA	13	0.2	0.304	0.243	0.706	0.456	0.250
2VRM_chainB	13	0.2	0.304	0.243	0.706	0.456	0.250
2XFP_chainB	13	0.135	0.296	0.196	0.923	0.528	0.395
2XFP_chainA	13	0.135	0.296	0.196	0.922	0.527	0.395
1OJA_chainA	13	0.135	0.296	0.196	0.922	0.526	0.396
1OJA_chainB	13	0.135	0.296	0.196	0.922	0.526	0.396
2BK5_chainA	13	0.135	0.296	0.196	0.922	0.526	0.396
2BK5_chainB	13	0.135	0.296	0.196	0.922	0.526	0.396
2BYB_chainA	13	0.133	0.276	0.208	0.807	0.641	0.166
2BYB_chainB	13	0.133	0.276	0.208	0.804	0.638	0.166
2VRL_chainA	13	0.231	0.273	0.229	0.744	0.411	0.333
2VRL_chainB	13	0.231	0.273	0.229	0.744	0.411	0.333
6FVZ_chainB	13	0.2	0.265	0.143	1.153	0.821	0.332
6FVZ_chainA	13	0.2	0.265	0.143	1.150	0.821	0.330
6FW0_chainA	13	0.225	0.265	0.18	1.148	0.816	0.332
6FWC_chainA	13	0.256	0.265	0.18	1.147	0.817	0.330
6FWC_chainB	13	0.256	0.265	0.18	1.147	0.817	0.330
6FW0_chainB	13	0.225	0.265	0.18	1.141	0.806	0.336
2C64_chainB	13	0.135	0.257	0.172	0.889	0.723	0.166

4A79_chainA	**13**	0.184	0.242	0.164	0.781	0.564	0.217
4A79_chainB	13	0.184	0.242	0.164	0.758	0.551	0.207
2C67_chainB	13	0.111	0.241	0.167	0.770	0.570	0.200
2C67_chainA	13	0.111	0.241	0.167	0.769	0.570	0.200
2C66_chainB	13	0.161	0.235	0.158	0.880	0.737	0.143
2C66_chainA	13	0.132	0.235	0.158	0.863	0.738	0.125
4A7A_chainA	13	0.132	0.235	0.161	0.807	0.569	0.239
4A7A_chainB	13	0.132	0.235	0.161	0.745	0.496	0.249
2VZ2_chainA	13	0.304	0.226	0.163	0.944	0.559	0.385
2VZ2_chainB	13	0.304	0.226	0.163	0.941	0.556	0.385
3PO7_chainB	13	0.12	0.226	0.157	0.880	0.605	0.276
3PO7_chainA	13	0.12	0.226	0.157	0.880	0.604	0.276
2C65_chainB	13	0.16	0.225	0.154	0.874	0.675	0.199
2C65_chainA	13	0.16	0.225	0.154	0.849	0.650	0.199
2BK4_chainA	13	0.133	0.219	0.151	0.845	0.679	0.167
2BK4_chainB	13	0.133	0.219	0.151	0.845	0.679	0.167
2XFQ_chainA	13	0.133	0.219	0.151	0.845	0.679	0.167
1S2Q_chainA	13	0.133	0.219	0.151	0.844	0.677	0.167
1S2Q_chainB	13	0.133	0.219	0.151	0.844	0.677	0.167
2XFQ_chainB	13	0.133	0.219	0.151	0.844	0.677	0.167
3ZYX_chainB	**13**	0.108	0.214	0.125	0.958	0.791	0.167
3ZYX_chainA	13	0.108	0.214	0.125	0.925	0.759	0.167
2V60_chainA	13	0.313	0.211	0.154	1.020	0.752	0.269
2V60_chainB	13	0.313	0.211	0.154	1.020	0.752	0.269
5MRL_chainB	13	0.147	0.207	0.13	0.747	0.580	0.167
1S3B_chainA	13	0.083	0.206	0.17	0.890	0.690	0.200
1S3B_chainB	13	0.083	0.206	0.17	0.871	0.671	0.200
2V61_chainA	13	0.256	0.205	0.149	0.879	0.705	0.174
2V61_chainB	13	0.256	0.205	0.149	0.866	0.676	0.190
2C73_chainB	13	0.1	0.182	0.151	0.865	0.698	0.167
2C75_chainA	13	0.1	0.182	0.151	0.839	0.673	0.166
2C75_chainB	13	0.083	0.182	0.151	0.835	0.669	0.167
1S2Y_chainA	13	0.083	0.182	0.151	0.834	0.667	0.167
1S2Y_chainB	13	0.083	0.182	0.151	0.834	0.667	0.167
2C76_chainB	13	0.083	0.182	0.151	0.833	0.667	0.167
2C76_chainA	13	0.083	0.182	0.151	0.832	0.665	0.167
2C73_chainA	13	0.1	0.182	0.151	0.806	0.640	0.166
2C72_chainA	13	0.1	0.182	0.151	0.744	0.578	0.166
2C72_chainB	13	0.1	0.182	0.151	0.744	0.577	0.166
4CRT_chainA	13	0.104	0.179	0.125	0.867	0.724	0.143
4CRT_chainB	13	0.104	0.179	0.125	0.846	0.703	0.143
1S3E_chainA	13	0.098	0.171	0.123	0.850	0.725	0.124
1S3E_chainB	13	0.098	0.171	0.123	0.849	0.725	0.124
5MRL_chainA	13	0.12	0.156	0.096	0.308	0.279	0.029
2XFQ_chainA	**13**	0.108	0.107	0.111	0.871	0.649	0.222
2XFQ_chainB	13	0.108	0.107	0.111	0.871	0.649	0.222
2XFP_chainB3	13	0.108	0.107	0.111	0.869	0.648	0.221
2XCG_chainB	13	0.108	0.107	0.111	0.869	0.647	0.222
2XCG_chainA	13	0.108	0.107	0.111	0.868	0.646	0.222
2XFN_chainB	13	0.108	0.107	0.111	0.868	0.646	0.223
2XFP_chainA3	13	0.108	0.107	0.111	0.868	0.646	0.222
2XFO_chainA	13	0.108	0.107	0.111	0.868	0.644	0.224

2XFN_chainA	13	0.108	0.107	0.111	0.865	0.641	0.224
2XFO_chainB	13	0.121	0.107	0.111	0.863	0.646	0.218
MAO-A ligand	***Compound ID***	***MACCSfp score***	***ECFP4fp score***	***ECFP6fp score***	***Tanimoto Combo***	***Shape Tanimoto***	***Color Tanimoto***
**2Z5X**	13	**0.135**	**0.219**	**0.143**	**0.831**	**0.705**	**0.126**
2Z5Y	**13**	0.135	0.219	0.143	0.839	0.713	0.126
2BXR_chainB	13	0.14	0.194	0.136	0.925	0.759	0.166
2BXS_chainB	13	0.14	0.194	0.136	0.854	0.687	0.166
2BXS_chainA	13	0.14	0.194	0.136	0.835	0.668	0.166
2BXR_chainA	13	0.14	0.194	0.136	0.831	0.665	0.166

As for MAO-A, the selection of the most suitable crystal structure (PDB code 2Z5X), was guided by visual inspection of the predicted 3D ligand-based alignments (Figure A). In fact, none of the reported crystallographic MAO-A ligands resulted to be similar to **13** according to employed 2D similarity indexes, the evaluated similarities being below commonly accepted thresholds (Table A).[15] Interestingly, these crystal structures have already been widely used in previous molecular modelling studies involving these targets.[16,17] Moreover, in the case of MAO-B, the selected crystal structure is in complex with the same ligand (safinamide)[13] that was used as a control in the *in vitro* assays.

Figure A: Predicted 3D shape-based alignments of 13 with the HRM crystallographic ligand (PDB ID 2Z5X).

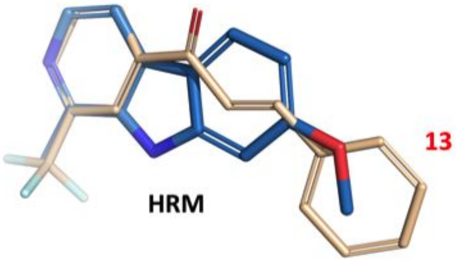

Structure-based calculations. Docking calculations were performed into the 2V5X (MAO-A) and 2V5Z (MAO-B) crystal structures by using the Glide software.[18,19] In particular, receptor grids (outer box of 10 Å × 10 Å × 10 Å) were firstly built around the co-crystallized ligands by using the default settings. The flavin adenine dinucleotide (FAD) cofactor and conserved water molecules were considered as components of the MAO binding sites during the structure-based

calculations. Then, redocking of the HRM (MAO-A) and SAG (MAO-B) ligands into their parent receptors was performed to assess the posing performance of the *in silico* models, obtaining root mean square deviation (RMSD) values below 2 Å. Afterwards, **13**, which resulted to be the most active compound of the investigated series, was docked into the validated MAO-A and MAO-B models, and the predicted binding modes (two poses with the best docking scores for each complex) were visually inspected. Docking complexes obtained with Glide were also post-processed with BEAR (Binding Estimation After Refinement), an *in-house* developed post-docking processing procedure, which consists of three steps based on molecular mechanics (MM) minimization and molecular dynamics cycles for the complex refinement, and the application of the MM-PBSA and MM-GBSA methods for binding free energy estimations.[20] Further details about the BEAR procedure, which has extensively demonstrated to be suitable for correcting and overcoming limitations of docking procedures, can be found in references.[21-23]

***In silico* ADME predictions.** *In silico* ADME (absorption, distribution, metabolism and excretion) predictions were performed to evaluate the drug-likeness and blood-brain-barrier permeability of compound **13**, which resulted to be the best candidate of the series. To this aim, the chalcone inhibitor was manually drawn in Maestro of the Schrodinger suite and prepared with the *LigPrep* software,[6,7] with default settings. Then, the prepared structure was analysed through the use of *QikProp* software available in the Schrödinger suite (release 2018-3).[24] Default settings were used in the ADME analyses, which allowed evaluating more than fifty different properties for compound **13**, including drug-likeness and molecules descriptors. The results of the analyses are reported in Table S1.

X-ray crystallography. Recombinant hMAO-B were expressed in *Pichia pastoris* and purified as previously described.[25] A protein sample was gel-filtered by Superdex200 (GE Healthcare) in 50 mM potassium phosphate pH 7.5, 8.5 mM Zwittergent 3-12. Fractions corresponding to a single peak eluted from the column were concentrated by Amicon30K (Millipore) up to about 2 mg mL^{-1} and co-crystallized with each chalcone inhibitor (**13** and **14**) by the sitting-drop vapour diffusion method following published protocols.[26] X-ray diffraction data were collected on crystals flash-cooled in liquid nitrogen (100 K, in mother liquor solution with 18% (v/v) glycerol added) using the beamlines of the Swiss Light Source in Villigen (Switzerland) and European Synchrotron Radiation Facility in Grenoble (France). Data processing and scaling (Table S3) were carried out using XDS[27] and the CCP4 package.[28] The coordinates of hMAO-B in complex with safinamide (PDB code 2V5Z,[13] was used as initial model after removing water and inhibitor molecules. Model building and analysis were performed by Coot,[29] whereas the program REFMAC5[30] was used for structure refinement. Figures were generated by Pymol.[31]

References

(1) Zhou, M.; Panchuk-Voloshina, N. A one-step fluorometric method for the continuous measurement of monoamine oxidase activity. *Anal. Biochem*. **1997**, *253*, 169-174.

(2) Di Paolo, M. L.; Christodoulou, M. S.; Calogero, A. M.; Pinzi, L.; Rastelli, G.; Passarella, D.; Cappelletti, G.; Dalla Via, L. 2-Phenyloxazole-4-carboxamide as a scaffold for selective inhibition of human monoamine oxidase B. *ChemMedChem* **2019**, *14*, 1641-1652.

(3) Madhavi Sastry, G.; Adzhigirey, M.; Day, T.; Annabhimoju, R.; Sherman, W. Protein and ligand preparation: parameters, protocols, and influence on virtual screening enrichments. *J. Comput. Aided Mol. Des.* **2013**, *27*, 221-234.

(4) Schrödinger Release 2018-3: Protein Preparation Wizard; Epik, Schrödinger, LLC, New York, NY, 2016; Impact, Schrödinger, LLC, New York, NY, **2016**; Prime, Schrödinger, LLC, New York, NY, 2018.

(5) Distinto, S.; Yáñez, M.; Alcaro, S.; Cardia, M. C.; Gaspari, M.; Sanna, M. L.; Meleddu, R.; Ortuso, F.; Kirchmair, J.; Markt, P.; Bolasco, A.; Wolber, G.; Secci, D.; Maccioni, E. Synthesis and biological assessment of novel 2-thiazolylhydrazones and computational analysis of their recognition by monoamine oxidase B. *Eur. J. Med. Chem.* **2012,** *48*, 284-295.

(6) Schrödinger Release 2018-3: Maestro, Schrödinger, LLC, New York, NY, **2018**.

(7) Schrödinger Release 2018-3: LigPrep, Schrödinger, LLC, New York, NY, **2018**.

(8) OMEGA 3.1.1.2: OpenEye Scientific Software, Santa Fe, NM, USA. http://www.eyesopen.com, **2019**.

(9) Hawkins, P. C. D.; Skillman, A. G.; Warren, G. L.; Ellingson, B. A.; Stahl, M. T. Conformer generation with OMEGA: algorithm and validation using high quality structures from the protein databank and Cambridge structural database. *J. Chem. Inf. Model*. **2010,** *50,* 572-584.

(10) ROCS 3.3.1.2: OpenEye Scientific Software, Santa Fe, NM. http://www.eyesopen.com.

(11) Hawkins, P. C. D.; Skillman, A. G.; Nicholls, A. Comparison of shape-matching and docking as virtual screening tools. *J. Med. Chem*. **2007**, *50*, 74-82.

(12) OpenEye Toolkits 2019.Apr.2 OpenEye Scientific Software, Santa Fe, NM. http://www.eyesopen.com.

(13) Binda, C.; Wang, J.; Pisani, L.; Caccia, C.; Carotti, A.; Salvati, P.; Edmondson, D.E.; Mattevi, A. Structures of human monoamine oxidase B Complexes with Selective noncovalent inhibitors: safinamide and coumarin analogs. *J. Med. Chem*. **2007**, *50*, 5848-5852.

(14) Son, S.-Y.; Ma, J.; Kondou, Y.; Yoshimura, M.; Yamashita, E.; Tsukihara, T.. Structure of human monoamine oxidase A at 2.2-Å resolution: the control of opening the entry for substrates/inhibitors. *Proc. Natl. Acad. Sci. USA* **2008,***105*, 5739-5744.

(15) Jasial, S.; Hu, Y.; Vogt, M.; Bajorath, J. Activity-relevant similarity values for fingerprints and implications for similarity searching. *F1000Research* **2016**, *5* Chem. Inf. Sci. 591.

(16) Shalaby, R.; Petzer, J. P.; Petzer, A.; Ashraf, U. M.; Atari, E.; Alasmari, F.; Kumarasamy, S.; Sari, Y.; Khalil, A. SAR and molecular mechanism studies of monoamine oxidase inhibition by selected chalcone analogs. *J. Enzyme Inhib. Med. Chem.* **2019**, *34*, 863-876.

(17) Morales-Camilo, N.; Salas, C. O.; Sanhueza, C.; Espinosa-Bustos, C.; Sepúlveda-Boza, S.; Reyes-Parada, M.; Gonzalez-Nilo, F.; Caroli-Rezende, M.; Fierro, A. Synthesis, biological

evaluation, and molecular simulation of chalcones and aurones as selective MAO-B inhibitors. *Chem. Biol. Drug Des.* **2015**, *85*, 685-695.

(18) Schrödinger Release 2018-3: Glide, Schrödinger, LLC, New York, NY, **2018**.

(19) Friesner, R. A.; Banks, J. L.; Murphy, R. B.; Halgren, T. A.; Klicic, J. J.; Mainz, D. T.; Repasky, M. P.; Knoll, E. H.; Shelley, M.; Perry, J. K.; Shaw, D. E.; Francis, P.; Shenkin, P. S. Glide: a new approach for rapid, accurate docking and scoring. 1. Method and assessment of docking accuracy. *J. Med. Chem*. **2004,** *47*, 1739-1749.

(20) Ben-Shalom, D. A.; Brozell, I.Y.; Cerutti, S. R.; Cheatham III, D. S.; Cruzeiro, T. E.; Darden, V. W. D.; Duke, T. A.; Ghoreishi, R. E.; Gilson, D.; Gohlke, M. K.; Goetz, H.; Greene, A. W.; Harris, D.; Homeyer, R.; Izadi, N.; Kovalenko, S.; Kurtzman, A.; Lee, T.; LeGrand, T. S.; Li, S.; Lin, P.; Liu, C.; Luchko, J.; Luo, T.; Mermelstein, R.; Merz, D. J.; Miao, K. M.; Monard, Y.; Nguyen, G.; Nguyen, C.; Omelyan, H.; Onufriev, I.; Pan, A.; Qi, F.; Roe, R.; Roitberg, D. R.; Sagui, A.; Schott-Verdugo, C.; Shen, S.; Simmerling, J.; Smith, C. I.; Salomon-Ferrer, J.; Swails, R.; Walker, J.; Wang, R. C.; Wei, J.; Wolf, H.; Wu, R.M.; Xiao, X.; York, L.; Kollman P.A. AMBER 2018, University of California, San Francisco. Available at: http://www.ambermd.org/.**2018**

(21) Rastelli, G.; Degliesposti, G.; Del Rio, A.; Sgobba, M. Binding estimation after refinement, a new automated procedure for the refinement and rescoring of docked ligands in virtual screening. *Chem. Biol. Drug Des*. **2009**, *73*, 283-286.

(22) Rastelli, G.; Rio, A. D.; Degliesposti; Sgobba, M. Fast and accurate predictions of binding free energies using MM-PBSA and MM-GBSA. *J. Comput. Chem*. **2010**, *31*, 797-810.

(23) Rastelli, G.; Pinzi, L. Refinement and rescoring of virtual screening results. *Front. Chem*. **2019**, 7.

(24) Schrödinger Release 2018-3: QikProp, Schrödinger, LLC, New York, NY, 2018.

(25) Newton-Vinson, P; Hubalek, F.; Edmondson, D. E. High-level expression of human liver monoamine oxidase B in *Pichia pastoris*. *Protein Expr. Purif.* **2000**, 20, 334-345.

(26) Binda, C.; Hubálek, F.; Li, M.; Herzig, Y.; Sterling, J.; Edmondson, D. E.; Mattevi, A. Crystal structures of monoamine oxidase B in complex with four inhibitors of the N-propargylaminoindan class. *J. Med. Chem*. **2004**, *47*, 1767-1774.

(27) Kabsch, W. XDS, *Acta Crystallogr. D*, **2010**, *66*, 125-132.

(28) The CCP4 suite: programs for protein crystallography, *Acta Crystallogr. D, Biol. Crystallogr*. **1994**, *50*, 760-763.

(29) Emsley, P.; Lohkamp, B.; Scott, W. G.; Cowtan, K. Features and development of Coot. *Acta Crystallogr. D, Biol. Crystallogr*. **2010,** *66*, 486-501.

(30) Murshudov, G. N.; Skubak, P.; Lebedev, A. A.; Pannu, N. S.; Steiner, R. A.; Nicholls, R. A; Winn, M. D.; Long, F.; Vagin, A. A. REFMAC5 for the refinement of macromolecular crystal structures. *Acta Crystallogr. D* **2011**, *67*, 355-367.

(31) V.S. The PyMOL Molecular Graphics System, LLC.

Table S1: ADME predictions of compound **13**. The calculated molecular descriptors and drug-like properties are within the recommended ranges reported in the reference manual of the QikProp software. Of note, compound 13 showed good agreement with Lipinski's rule of five ("*RuleOfFive*") and Jorgensen's rule of three ("*RuleOfThree*"), the number of violations evaluated being 0. Moreover, the compound showed favorable values of brain/blood partition coefficient ("*QPlogBB*"), binding to human serum albumin ("*QPlogKhsa*"), and apparent Caco-2 ("*QPPCaco*") and MDCK ("*QPPMDCK*") cell permeabilities.

Property	Value	Property	Value
**#stars**	0	**QPlogS**	-5.02
**#amine**	0	**CIQPlogS**	-4.731
**#amidine**	0	**QPlogHERG**	-5.761
**#acid**	0	**QPPCaco**	3710.501
**#amide**	0	**QPlogBB**	0.186
**#rotor**	4	**QPPMDCK**	8935.846
**#rtvFG**	1	**QPlogKp**	-0.745
**CNS**	1	**IP(eV)**	9.537
**mol_MW**	276.258	**EA(eV)**	1.062
**dipole**	1.567	**#metab**	1
**SASA**	527.217	**QPlogKhsa**	0.46
**FOSA**	18.999	**HumanOralAbsorption**	3
**FISA**	44.972	**PercentHumanOralAbsorption**	100
**PISA**	346.178	**SAfluorine**	117.068
**WPSA**	117.068	**SAamideO**	0
**volume**	877.395	**PSA**	27.117
**donorHB**	0	**#NandO**	1
**accptHB**	2	**RuleOfFive**	0
**dip^2/V**	0.003	**#ringatoms**	12
**ACxDN^.5/SA**	0	**#in34**	0
**glob**	0.841	**#in56**	12
**QPpolrz**	29.697	**#noncon**	0
**QPlogPC16**	8.256	**#nonHatm**	20
**QPlogPoct**	11.011	**RuleOfThree**	0
**QPlogPw**	4.777	**Jm**	0.475
**QPlogPo/w**	4.586		

Table S2: Binding free energy predictions (kcal·mol^{-1}) of **13** in MAO-B made with BEAR (Binding Estimation After Refinement).

	**13** - *a* binding mode	**13** - *b* binding mode
ΔG_{vacuo}	0	-1.5
ΔG_{solv}	0	-5.3
ΔG_{bind}	0	-6.8

Table S3. Data collection and refinement statistics for the crystal structures of human MAO-B in complex with **13** and **14**.

	**13**	**(14)**
Space group	C222	C222
Unit cell axes (Å)	*a* = 130.6	*a* = 131.3
	b = 221.8	*b* = 221.9
	c = 85.8	*c* = 86.1
Resolution (Å)	2.3	2.1
PDB code	7B0V	7B0Z
$R_{sym}{}^{a,b}$ (%)	18.0 (58.0)	18.4 (72.1)
$CC_{1/2}$ (%)	99.1 (87.6)	99.3 (81.1)
Completeness^{b} (%)	99.9 (100.0)	99.9 (100.0)
Unique reflections	55,710	73,620
Redundancy	7.3 (7.6)	6.7 (6.8)
I/σ^{b}	11.1 (3.6)	11.2 (3.1)
N° of non-hydrogen		
atoms protein/FAD	7928/2x53	7928/2x53
inhibitor	2x20	2x20
detergent^{c}/glycerol	26/2x6	26/2x6
water	454	714
Average B value for		
protein/inhibitor	17.0/30.5	19.3/38.9
atoms (Å^{2})		
$R_{cryst}{}^{b,d}$ (%)	16.7 (19.0)	16.1 (19.3)
$R_{free}{}^{b,d}$ (%)	18.8 (19.4)	18.8 (21.8)
Rms bond length (Å)	0.010	0.011
Rms bond angles (°)	1.62	1.60

^{a} $R_{sym}=\sum|I_i-\langle I\rangle|/\sum I_i$, where I_i is the intensity of ith observation and <I> is the mean intensity of the reflection.

^{b} Values in parentheses are for reflections in the highest resolution shell.

^{c} As in previous human MAO-B structures, one molecule of the Zwittergent 3-12 detergent (used in crystallization experiments) is partly visible in the electron density of each of the two protein monomers present in the asymmetric unit ^{d} $R_{cryst}=\sum|F_{obs}-F_{calc}|/\sum|F_{obs}|$ where F_{obs} and F_{calc} are the observed and calculated structure factor amplitudes, respectively. R_{cryst} and R_{free} were calculated using the working and test sets, respectively.

Figure S1: Binding mode a predicted for compound **13** into the 2V5Z crystal structure (MAO-B).

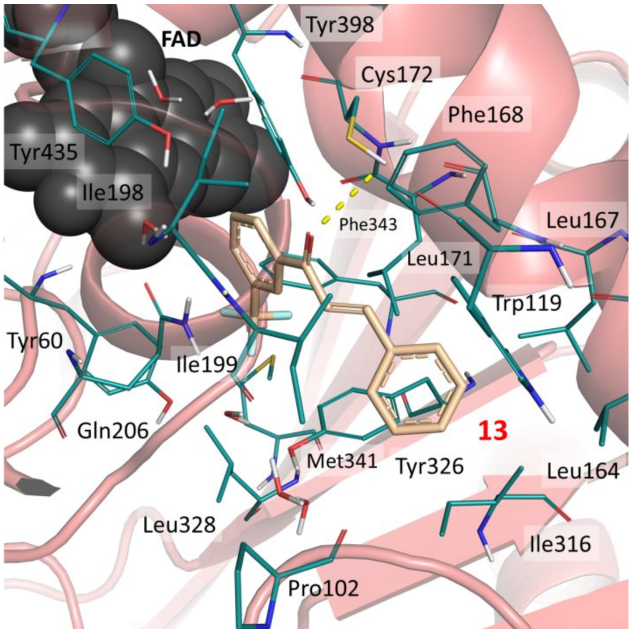

Figure S2: Binding mode predicted for compound **14** into the 2V5Z crystal structure (MAO-B).

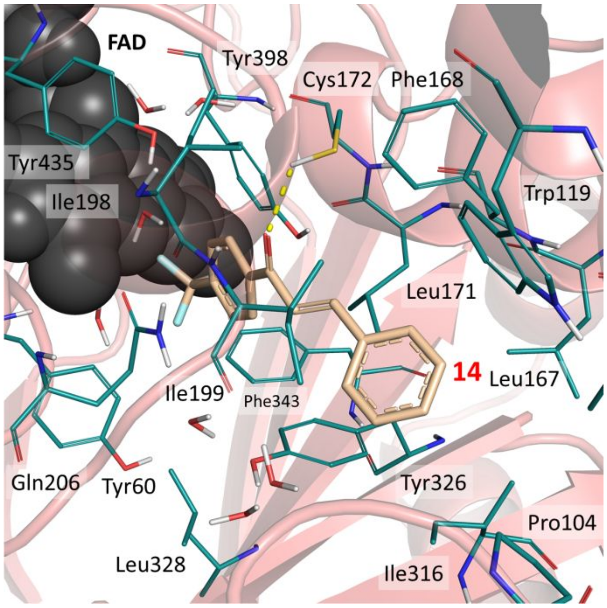

NMR Spectra of compounds 1-17

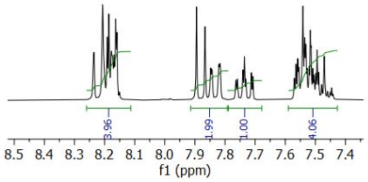

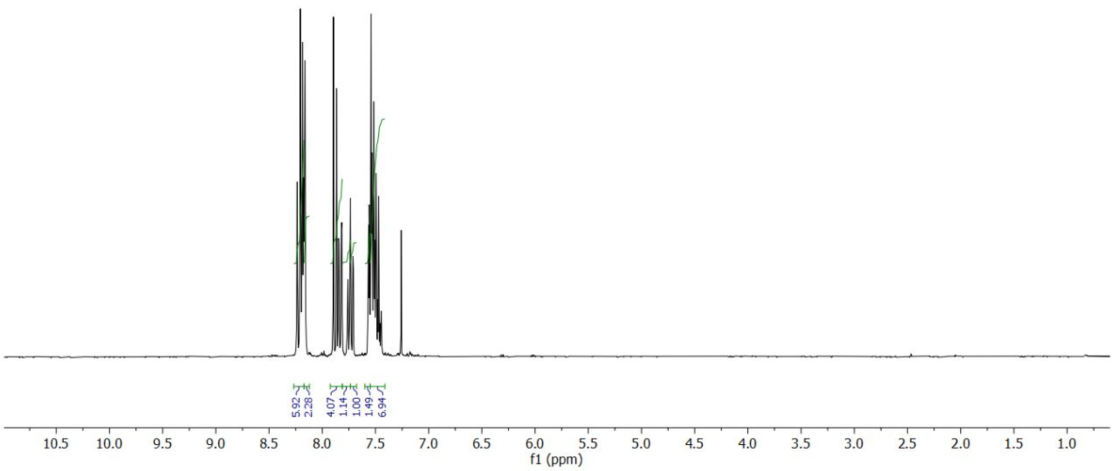

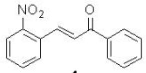

1

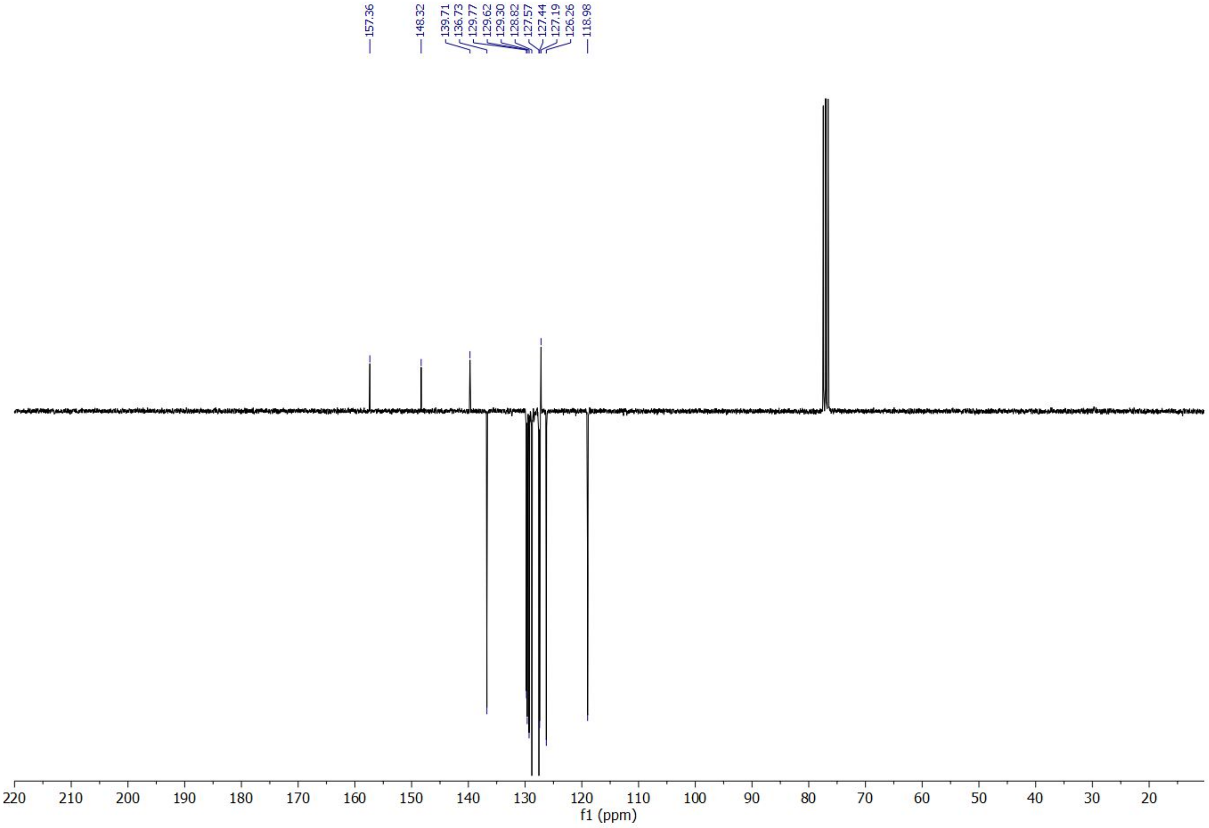

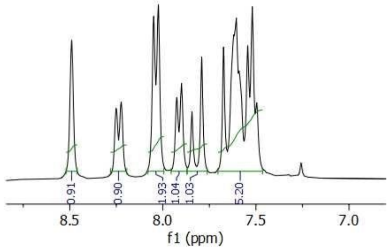
0.91
0.90
1.93
1.04
1.03
5.20
f1 (ppm)

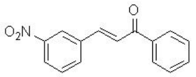
O
O_2N

2

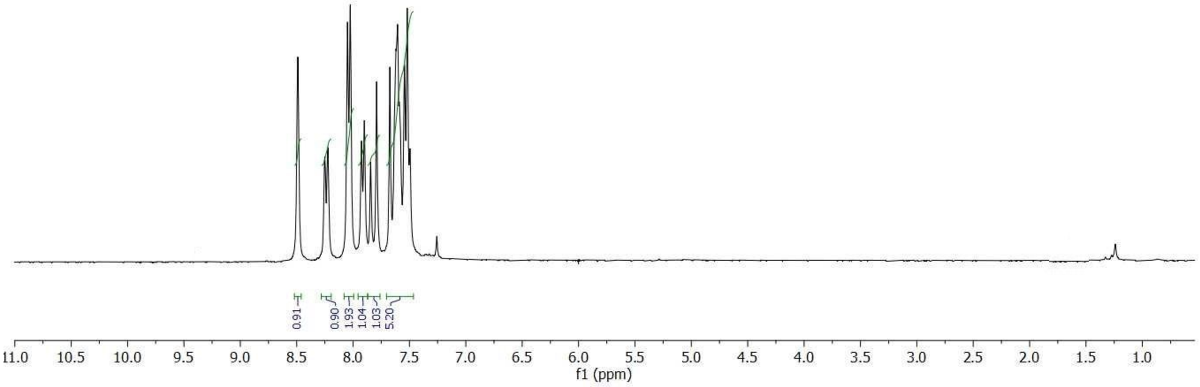
0.91
0.90
1.93
1.04
1.03
5.20
f1 (ppm)

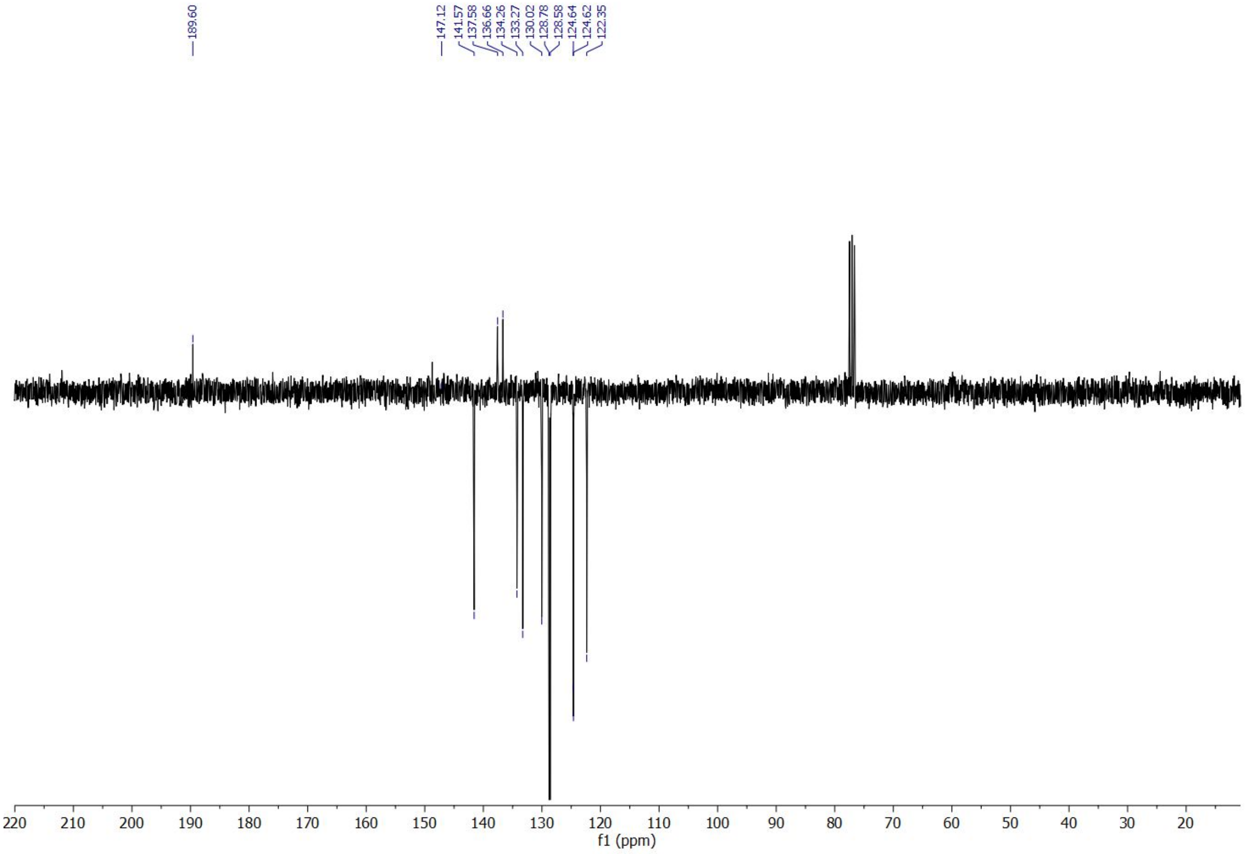
189.60
147.12
141.57
137.58
136.66
134.26
133.27
130.02
128.78
128.58
124.64
124.62
122.35
f1 (ppm)

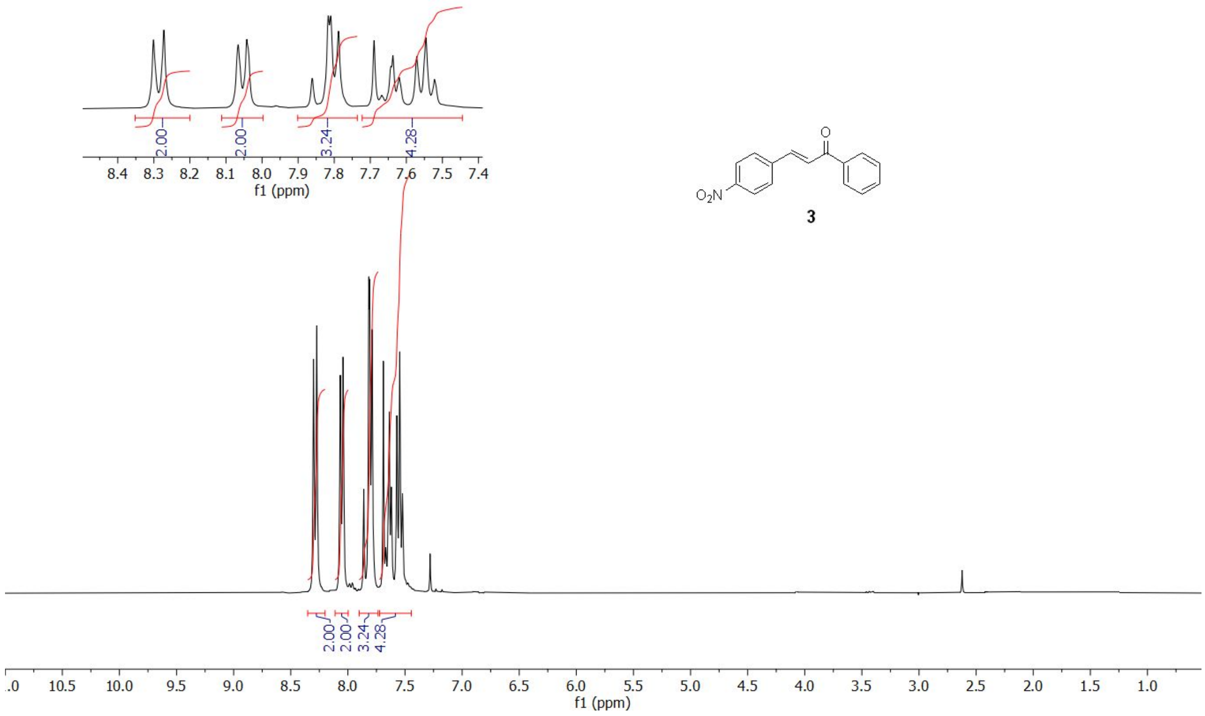
2.00
2.00
3.24
4.28
f1 (ppm)
O
O_2N

3

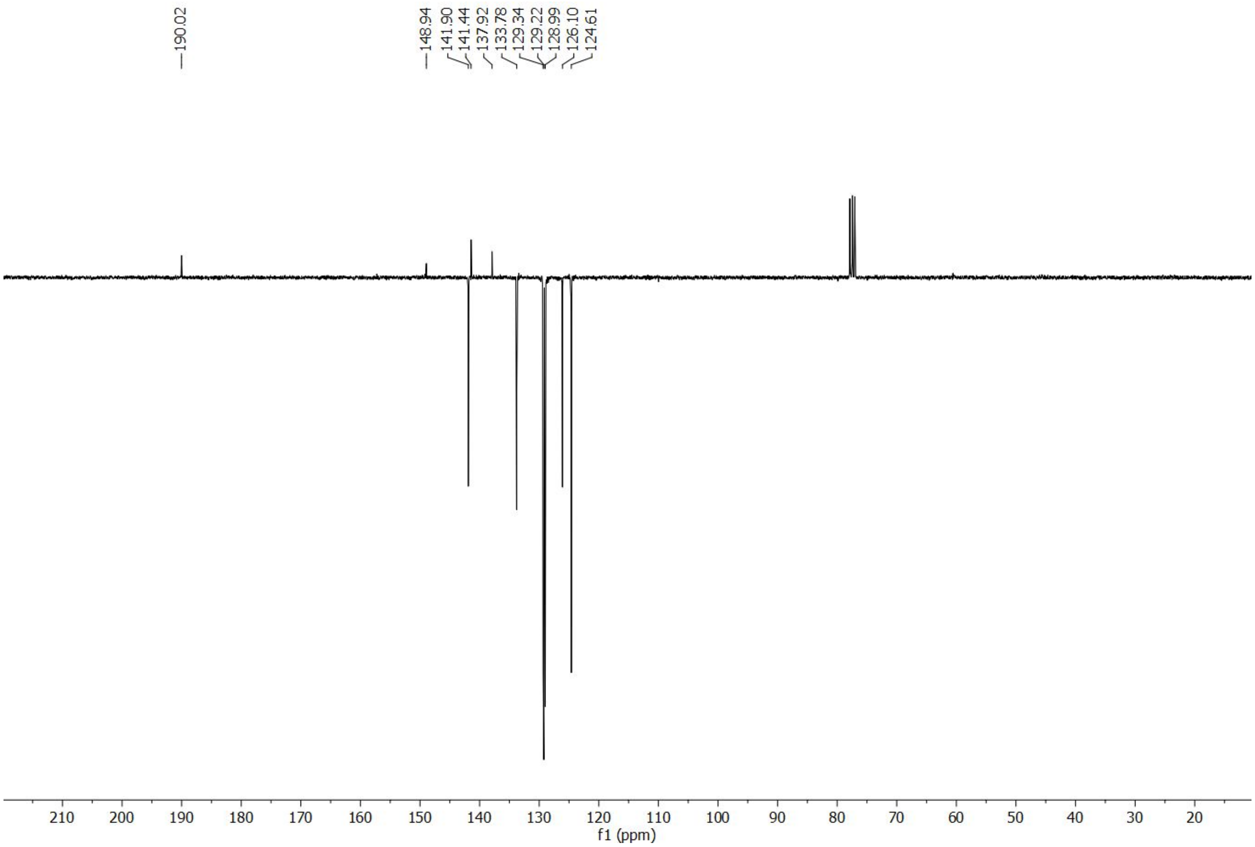
190.02
148.94
141.90
141.44
137.92
133.78
129.34
129.22
128.99
126.10
124.61
f1 (ppm)

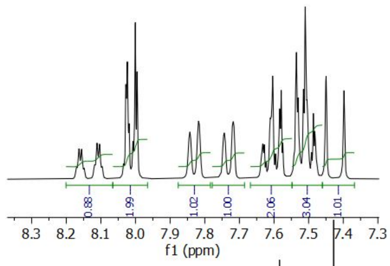
0.88
1.99
1.02
1.00
2.06
3.04
1.01
f1 (ppm)

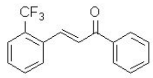
CF3
O

4

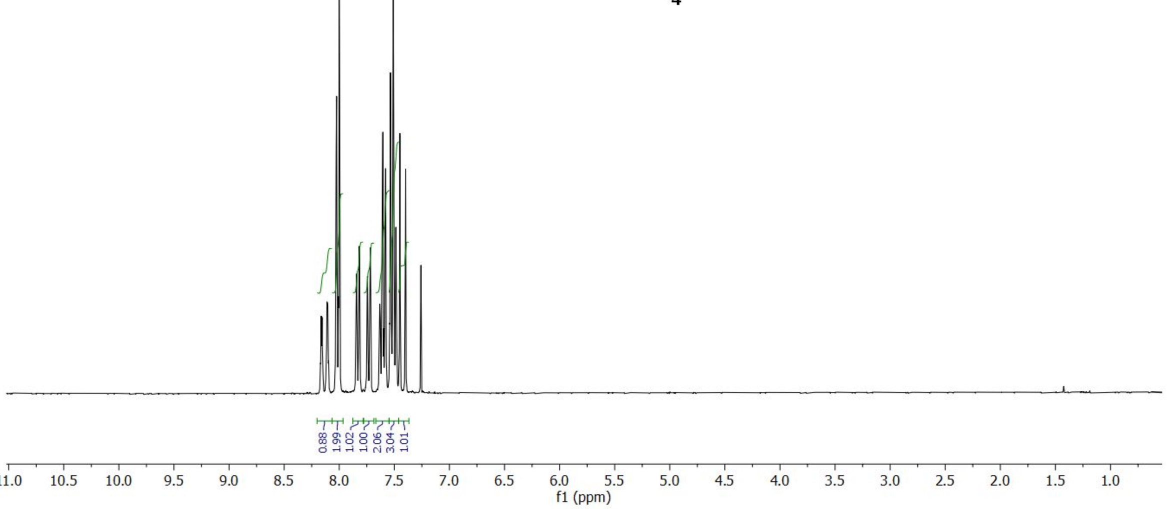
0.88
1.99
1.02
1.00
2.06
3.04
1.01
f1 (ppm)

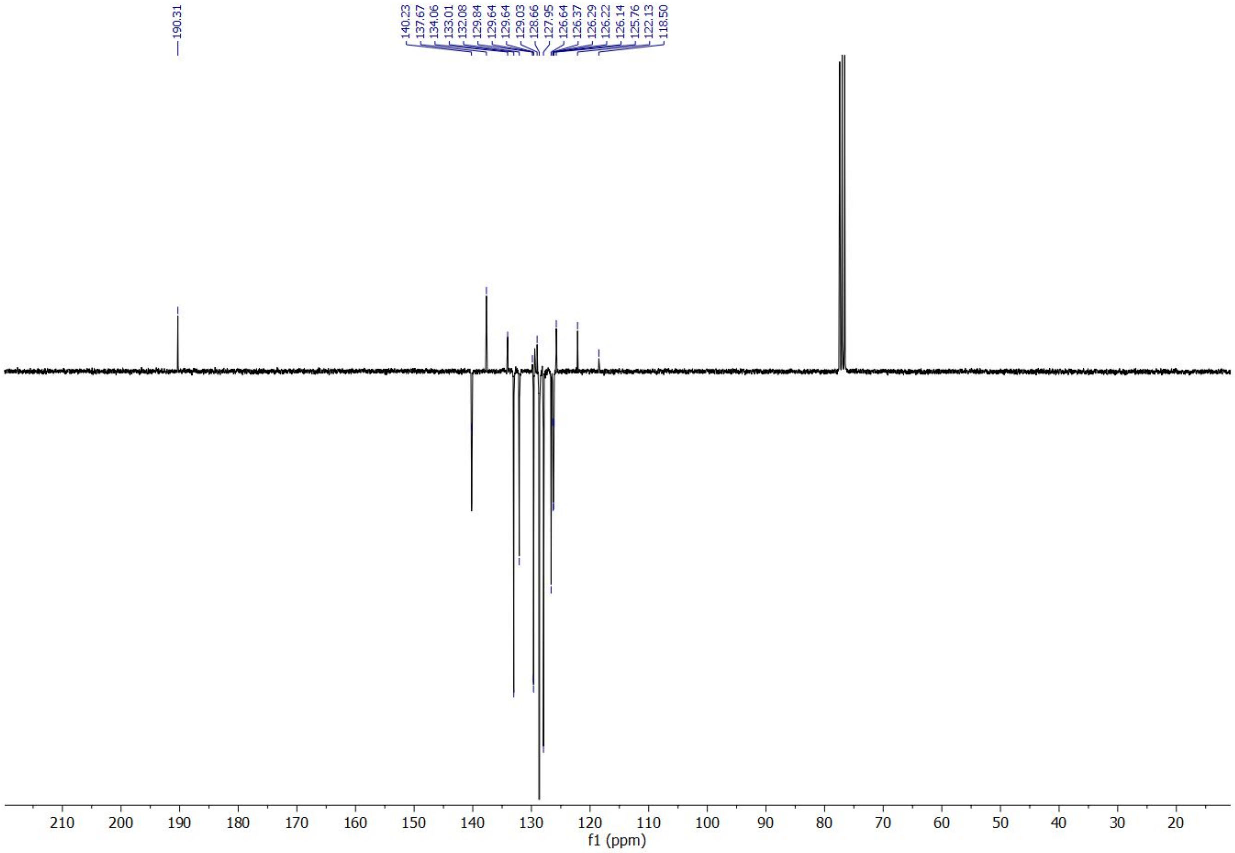
190.31
140.23
137.67
134.06
133.01
132.08
129.84
129.64
129.64
129.03
128.66
127.95
126.64
126.37
126.29
126.22
126.14
125.76
122.13
118.50
f1 (ppm)

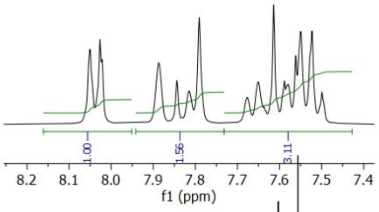
1.00
1.56
3.11
f1 (ppm)

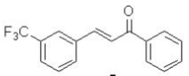
F_3C
O

5

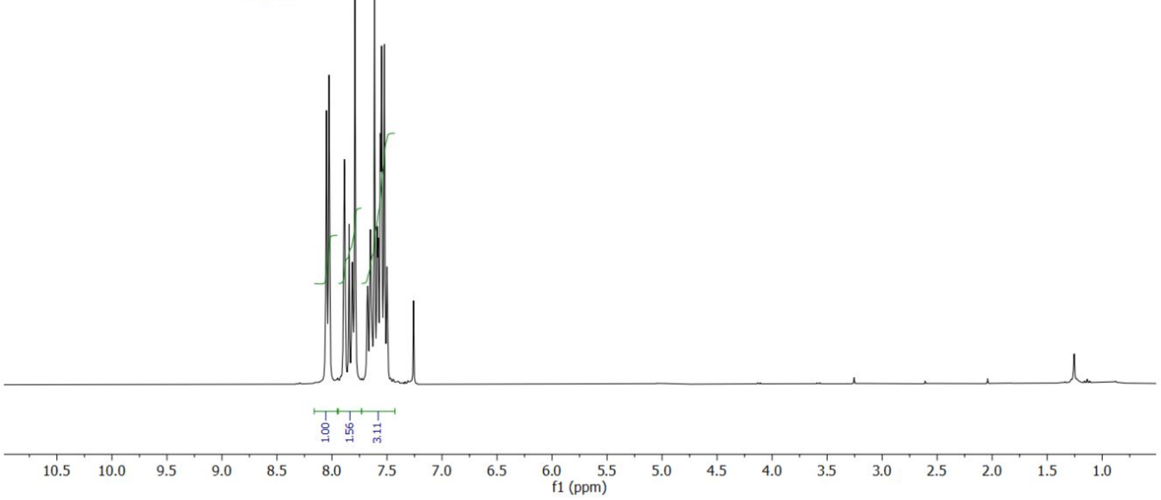
1.00
1.56
3.11
f1 (ppm)

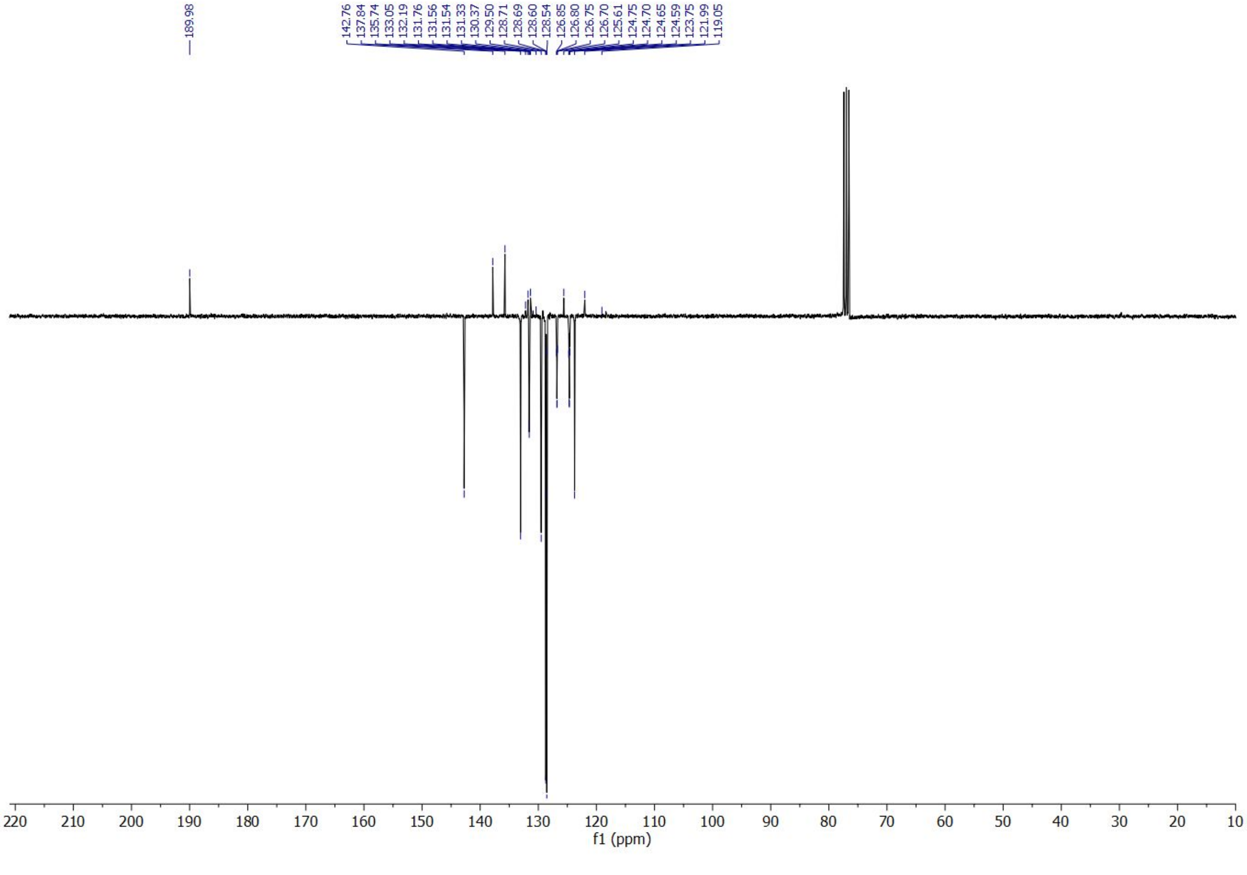
189.98
142.76
137.84
135.74
133.05
132.19
131.76
131.56
131.54
131.33
130.37
129.50
128.71
128.69
128.60
128.54
126.85
126.80
126.75
126.70
125.61
124.75
124.70
124.65
124.59
123.75
121.99
119.05
f1 (ppm)

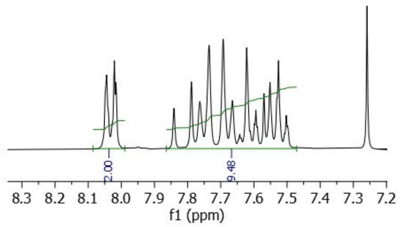
2.00
9.48
8.3 8.2 8.1 8.0 7.9 7.8 7.7 7.6 7.5 7.4 7.3 7.2
f1 (ppm)

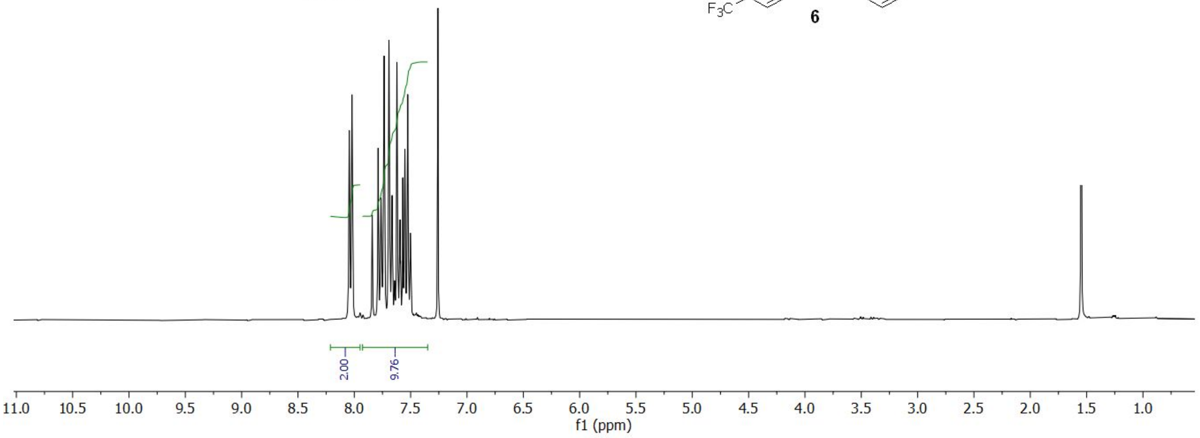
2.00
9.76
11.0 10.5 10.0 9.5 9.0 8.5 8.0 7.5 7.0 6.5 6.0 5.5 5.0 4.5 4.0 3.5 3.0 2.5 2.0 1.5 1.0
f1 (ppm)

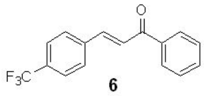
O
F3C

6

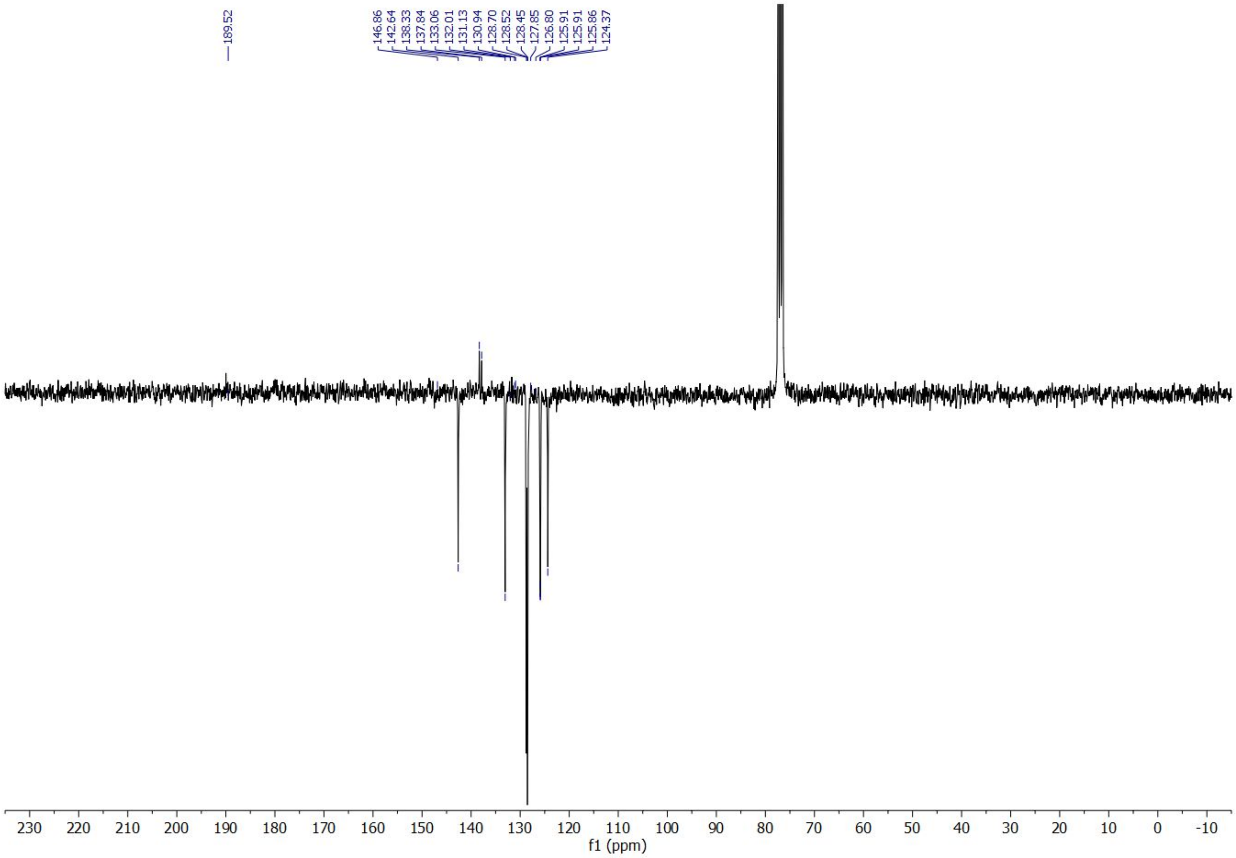
189.52
146.86
142.64
138.33
137.84
133.06
132.01
131.13
130.94
128.70
128.52
128.45
127.85
126.80
125.91
125.91
125.86
124.37
230 220 210 200 190 180 170 160 150 140 130 120 110 100 90 80 70 60 50 40 30 20 10 0 -10
f1 (ppm)

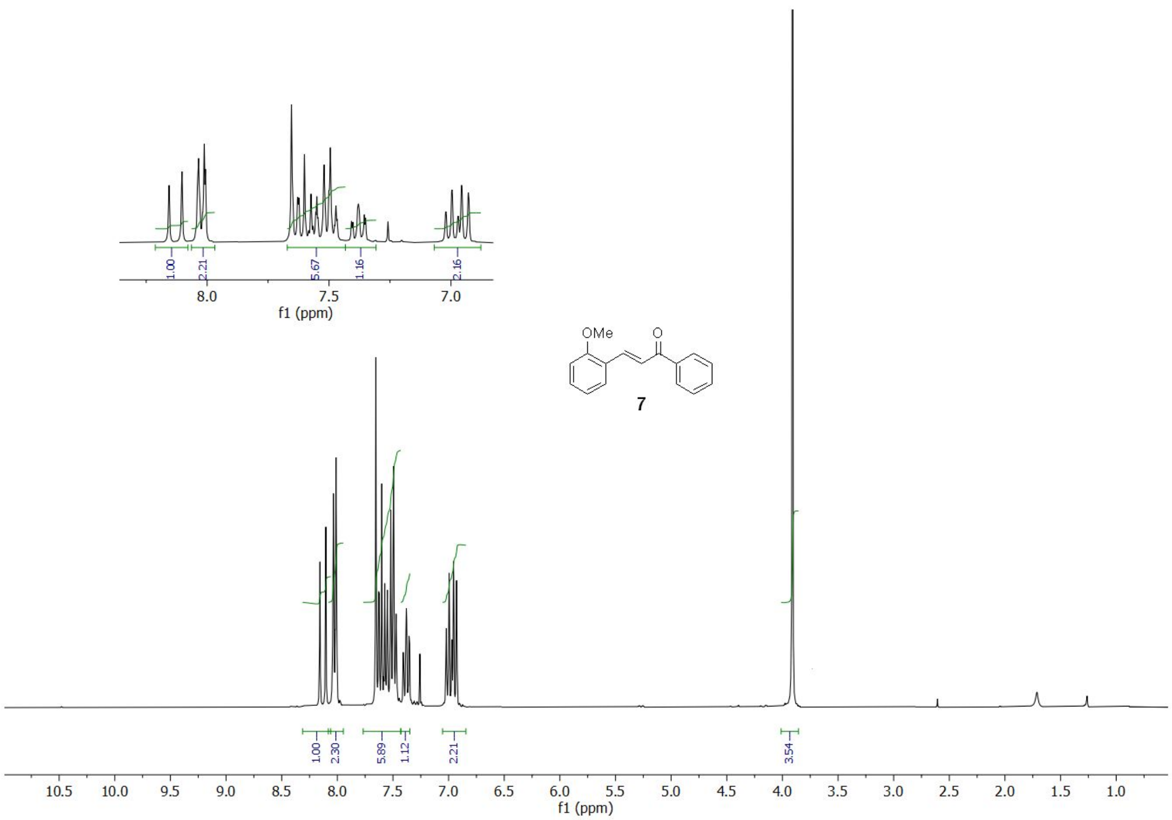
OMe
O
7
1.00
2.21
5.67
1.16
2.16
8.0
7.5
7.0
f1 (ppm)
1.00
2.30
5.89
1.12
2.21
3.54
10.5 10.0 9.5 9.0 8.5 8.0 7.5 7.0 6.5 6.0 5.5 5.0 4.5 4.0 3.5 3.0 2.5 2.0 1.5 1.0
f1 (ppm)

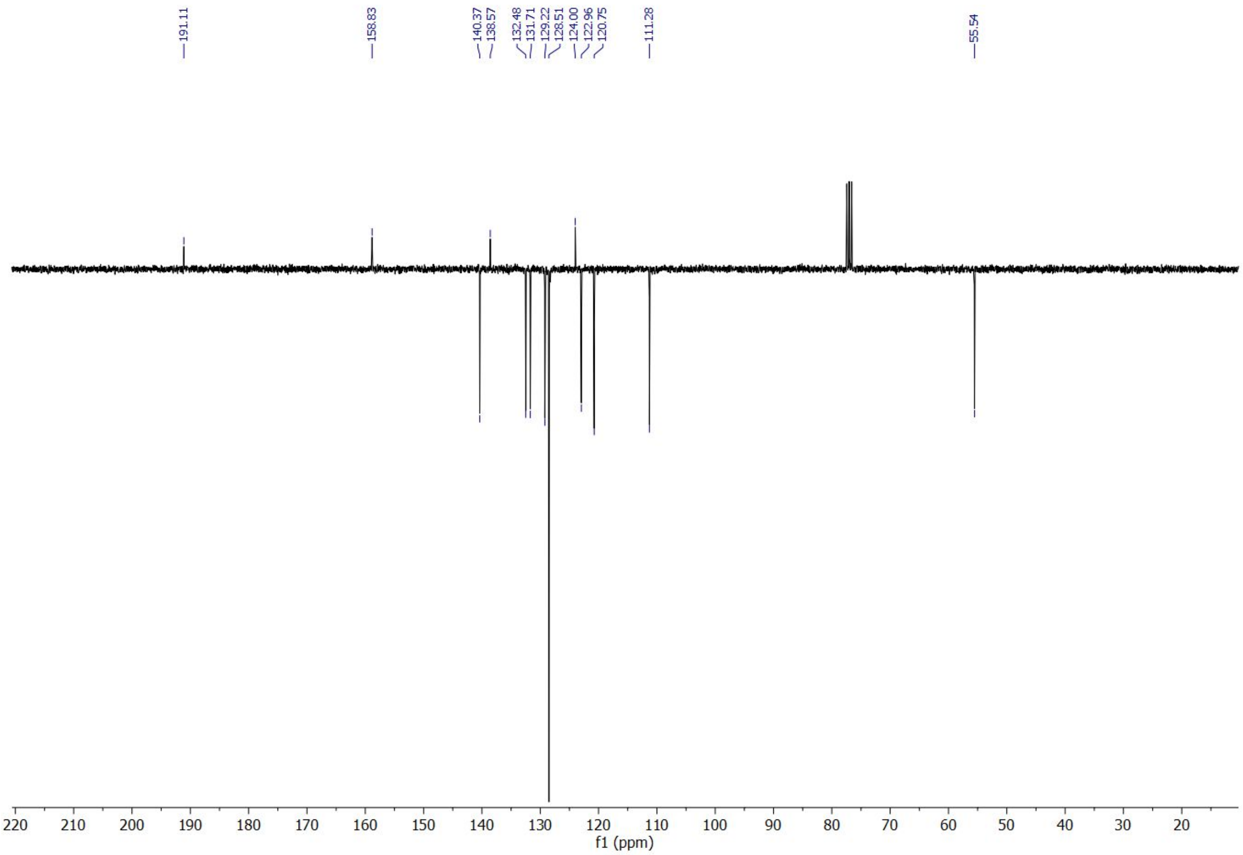
191.11
158.83
140.37
138.57
132.48
131.71
129.22
128.51
124.00
122.96
120.75
111.28
55.54
220 210 200 190 180 170 160 150 140 130 120 110 100 90 80 70 60 50 40 30 20
f1 (ppm)

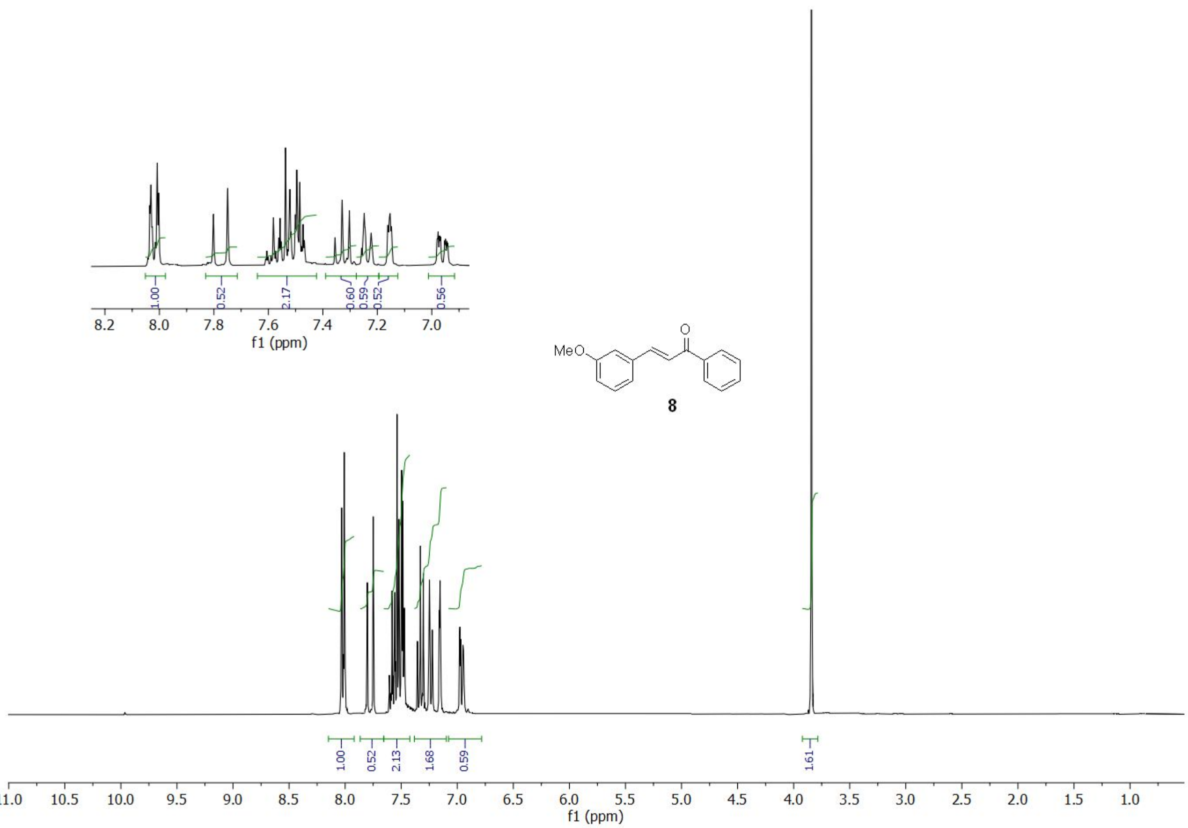
1.00
0.52
2.17
0.60
0.59
0.52
0.56
f1 (ppm)
MeO
O
8
1.00
0.52
2.13
1.68
0.59
1.61
f1 (ppm)

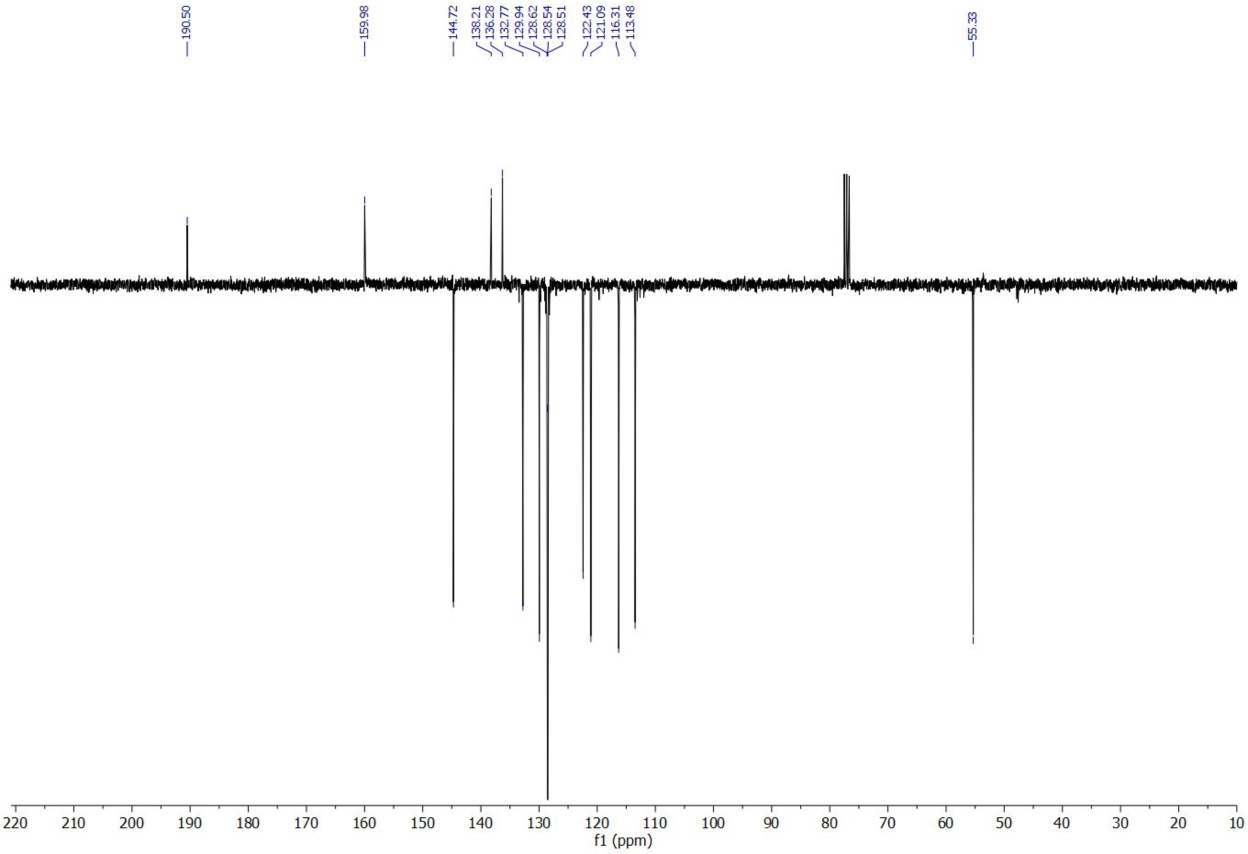
190.50
159.98
144.72
138.21
136.28
132.77
129.94
128.62
128.54
128.51
122.43
121.09
116.31
113.48
55.33
f1 (ppm)

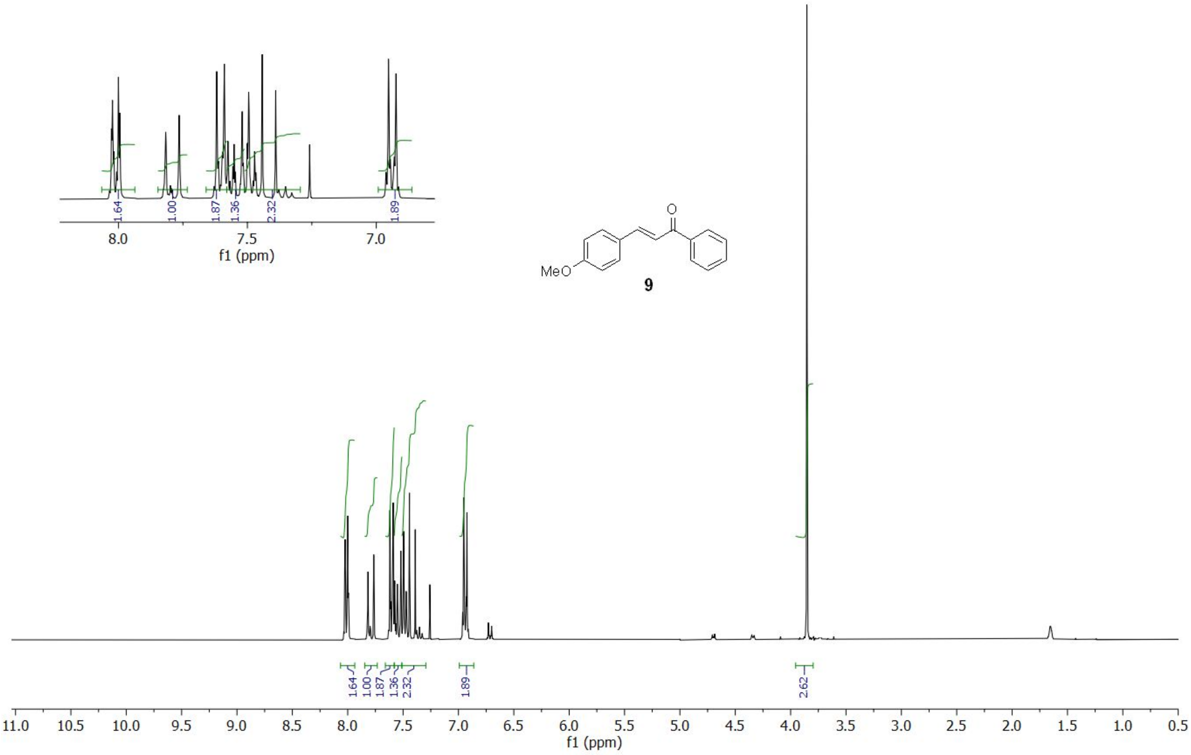
f1 (ppm)
1.64
1.00
1.87
1.36
2.32
1.89
2.62

9

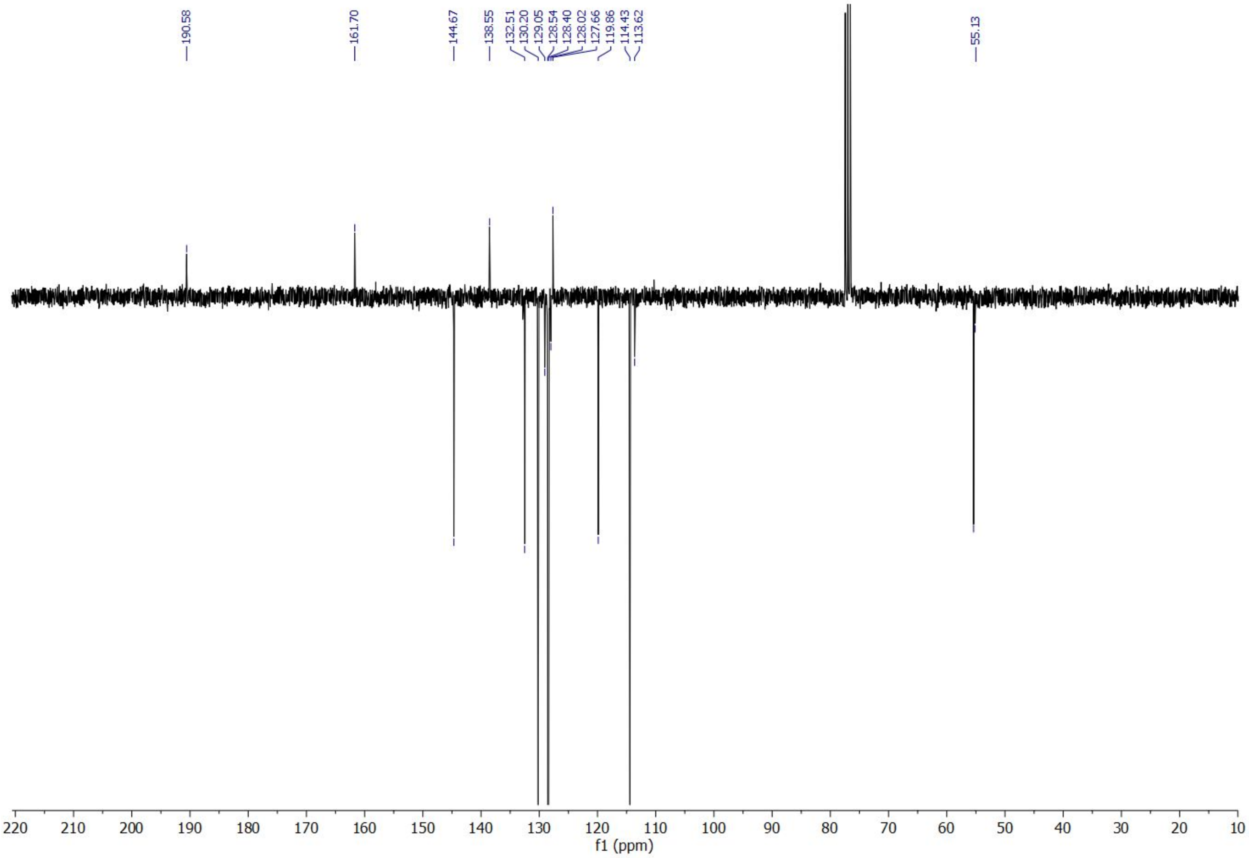
190.58
161.70
144.67
138.55
132.51
130.20
129.05
128.54
128.40
128.02
127.66
119.86
114.43
113.62
55.13
f1 (ppm)

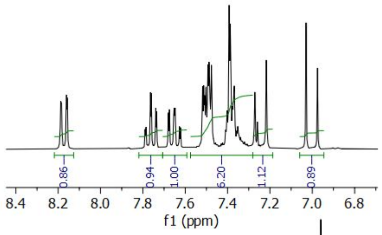
0.86
0.94
1.00
6.20
1.12
0.89
8.4 8.2 8.0 7.8 7.6 7.4 7.2 7.0 6.8
f1 (ppm)

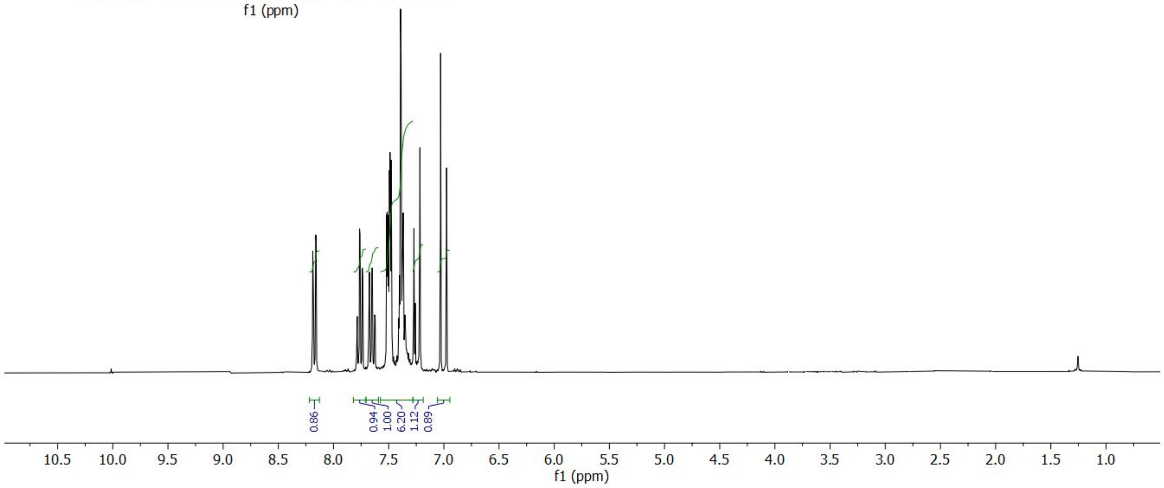
0.86
0.94
1.00
6.20
1.12
0.89
10.5 10.0 9.5 9.0 8.5 8.0 7.5 7.0 6.5 6.0 5.5 5.0 4.5 4.0 3.5 3.0 2.5 2.0 1.5 1.0
f1 (ppm)

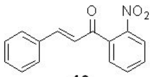
O
NO2

10

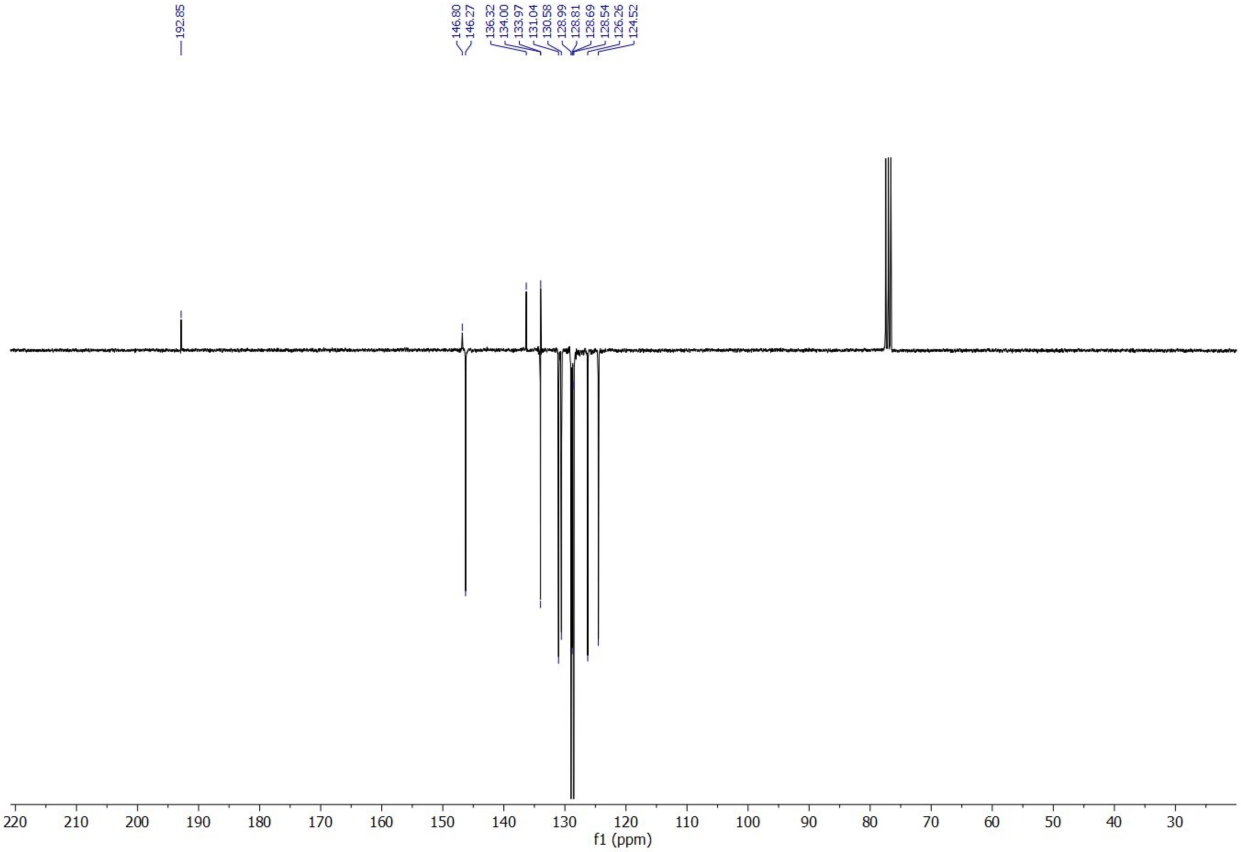
192.85
146.80
146.27
136.32
134.00
133.97
131.04
130.58
128.99
128.81
128.69
128.54
126.26
124.52
220 210 200 190 180 170 160 150 140 130 120 110 100 90 80 70 60 50 40 30
f1 (ppm)

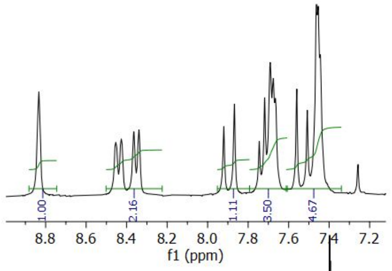
1.00
2.16
1.11
3.50
4.67
8.8 8.6 8.4 8.2 8.0 7.8 7.6 7.4 7.2
f1 (ppm)

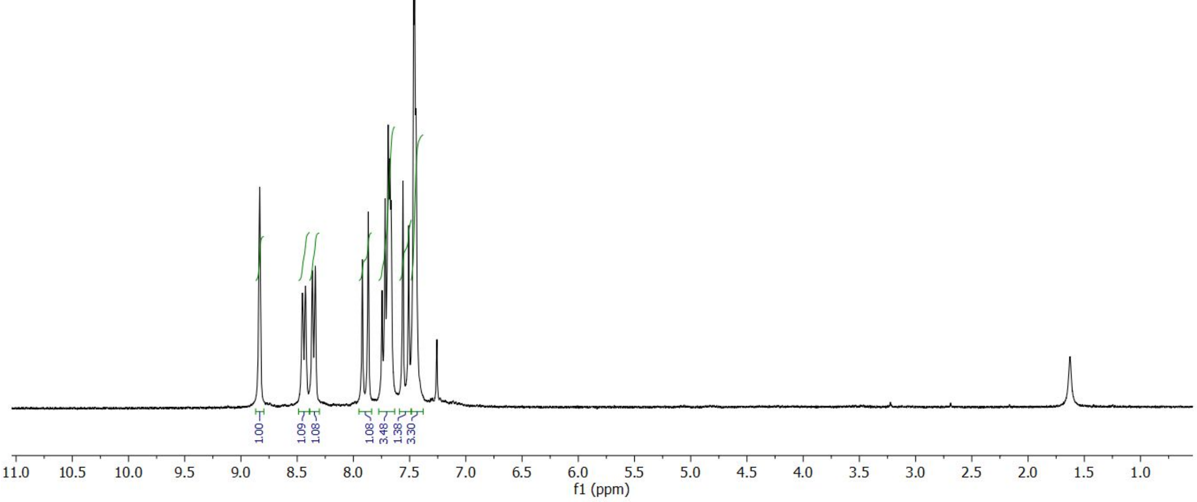
1.00
1.09
1.08
1.08
3.48
1.38
3.30
11.0 10.5 10.0 9.5 9.0 8.5 8.0 7.5 7.0 6.5 6.0 5.5 5.0 4.5 4.0 3.5 3.0 2.5 2.0 1.5 1.0
f1 (ppm)

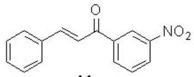
O
NO_2

11

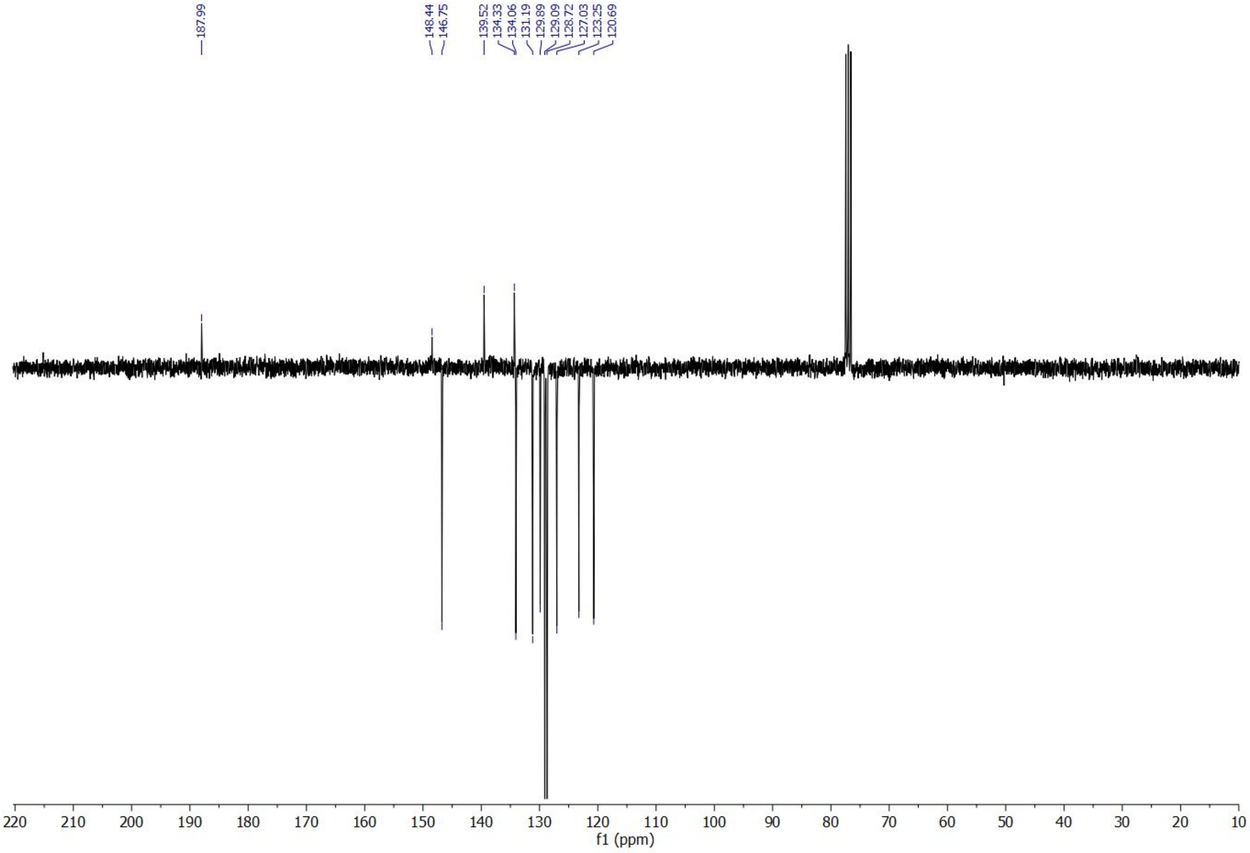
187.99
148.44
146.75
139.52
134.33
134.06
131.19
129.89
129.09
128.72
127.03
123.25
120.69
220 210 200 190 180 170 160 150 140 130 120 110 100 90 80 70 60 50 40 30 20 10
f1 (ppm)

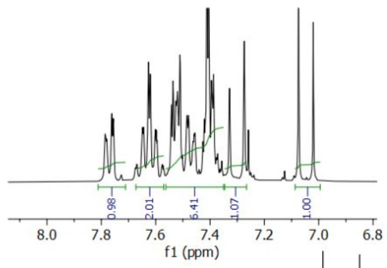
0.98
2.01
6.41
1.07
1.00
8.0 7.8 7.6 7.4 7.2 7.0 6.8
f1 (ppm)

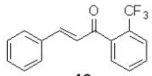
O
CF3

12

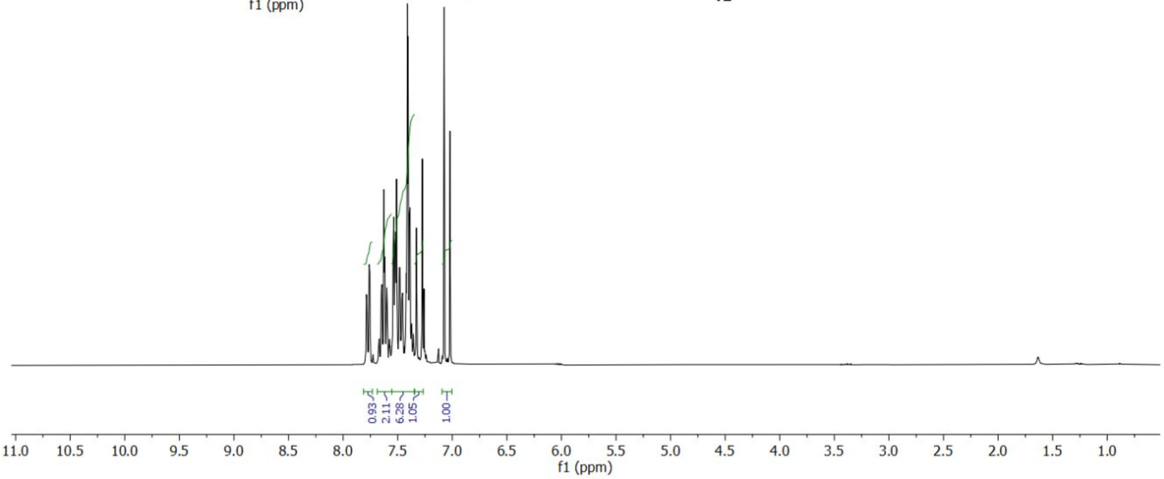
0.93
2.11
6.28
1.05
1.00
11.0 10.5 10.0 9.5 9.0 8.5 8.0 7.5 7.0 6.5 6.0 5.5 5.0 4.5 4.0 3.5 3.0 2.5 2.0 1.5 1.0
f1 (ppm)

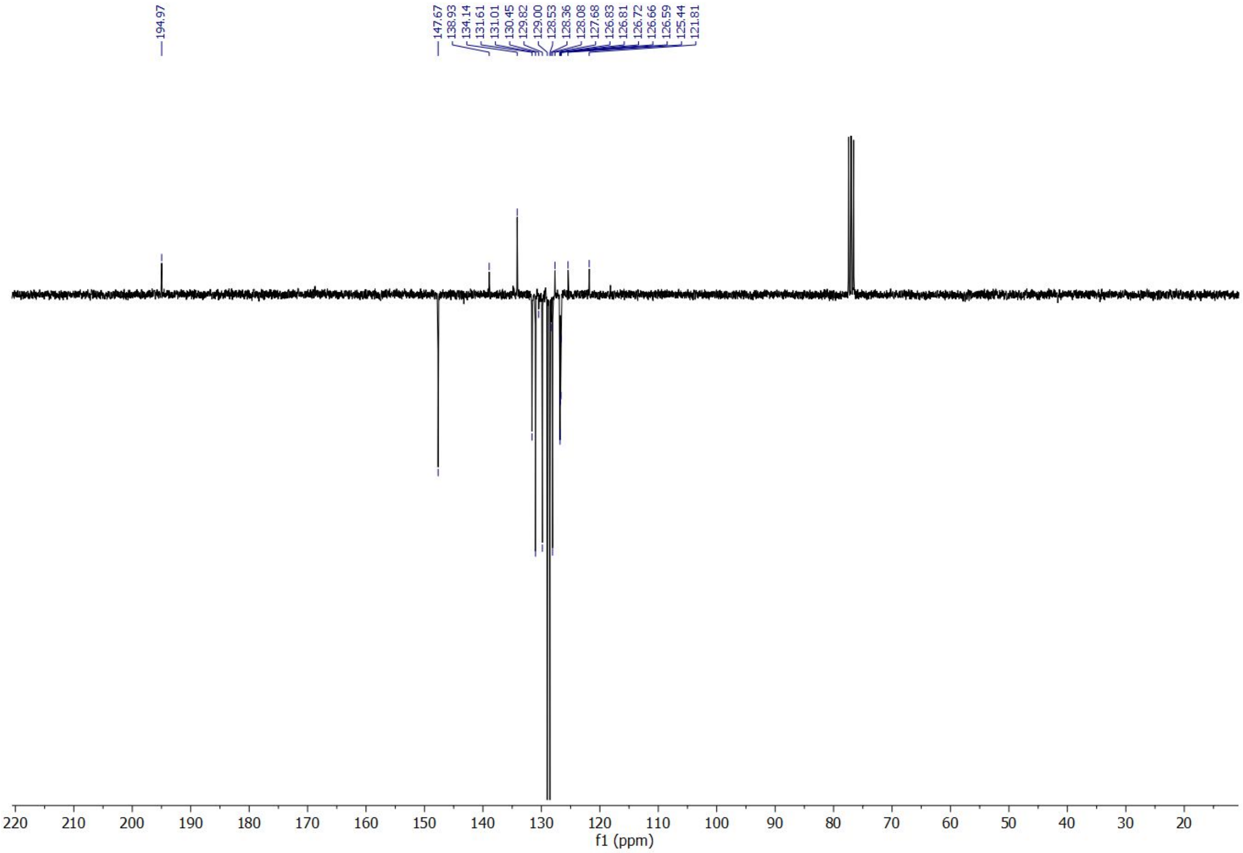
194.97
147.67
138.93
134.14
131.61
131.01
130.45
129.82
129.00
128.53
128.36
128.08
127.68
126.83
126.81
126.72
126.66
126.59
125.44
121.81
220 210 200 190 180 170 160 150 140 130 120 110 100 90 80 70 60 50 40 30 20
f1 (ppm)

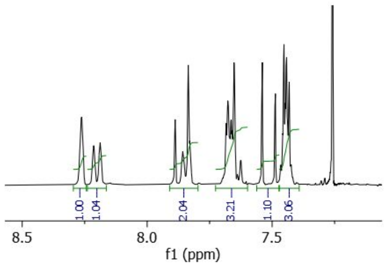

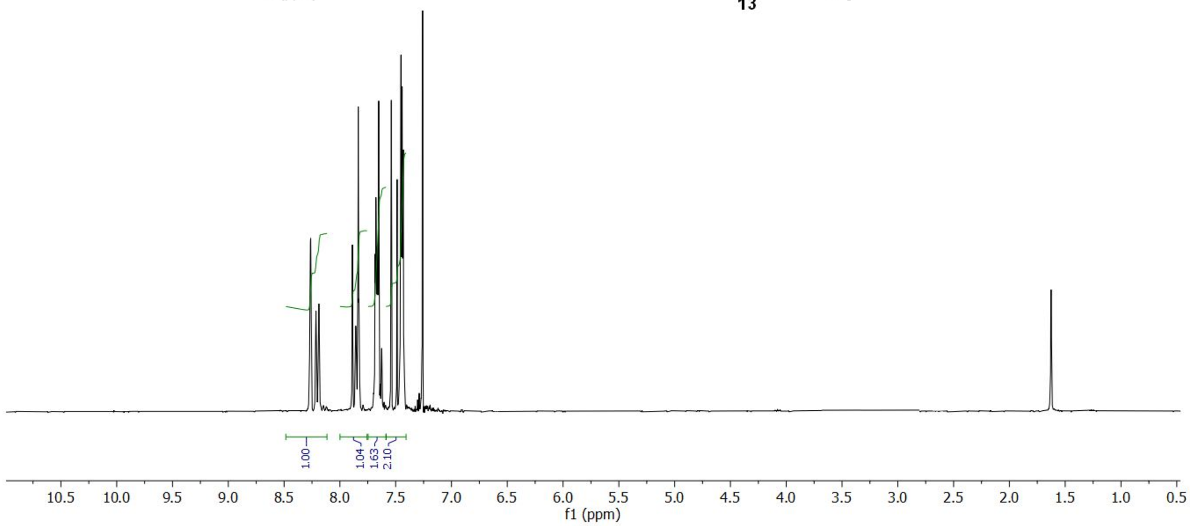
1.00
1.04
2.04
3.21
1.10
3.06
8.5
8.0
7.5
f1 (ppm)
1.00
1.04
1.63
2.10
10.5 10.0 9.5 9.0 8.5 8.0 7.5 7.0 6.5 6.0 5.5 5.0 4.5 4.0 3.5 3.0 2.5 2.0 1.5 1.0 0.5
f1 (ppm)

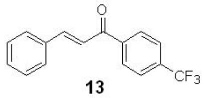
O
CF3

13

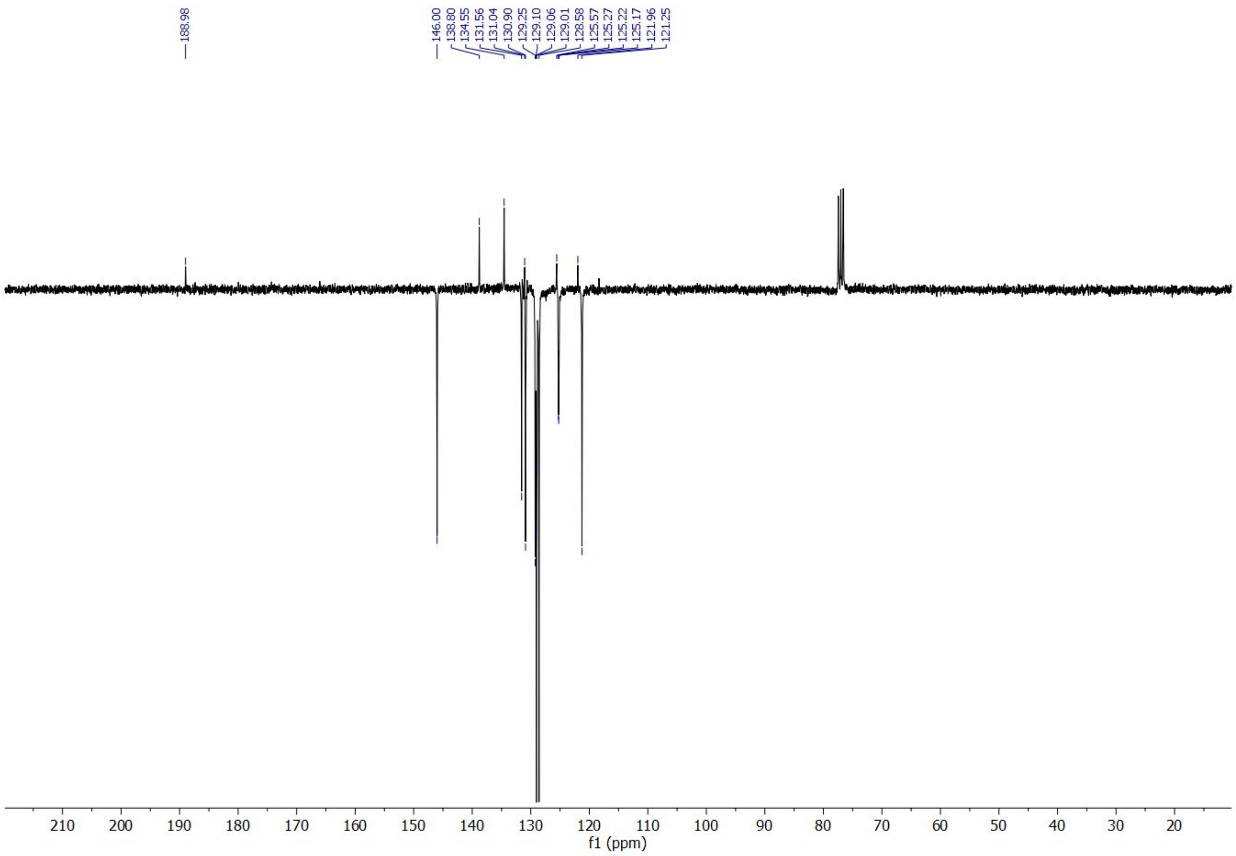
188.98
146.00
138.80
134.55
131.56
131.04
130.90
129.25
129.10
129.06
129.01
128.58
125.57
125.27
125.22
125.17
121.96
121.25
210 200 190 180 170 160 150 140 130 120 110 100 90 80 70 60 50 40 30 20
f1 (ppm)

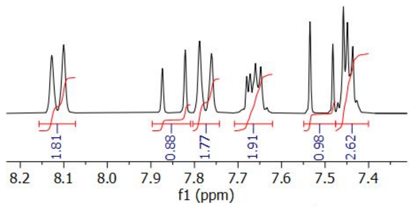
1.81
0.88
1.77
1.91
0.98
2.62
8.2 8.1 8.0 7.9 7.8 7.7 7.6 7.5 7.4
f1 (ppm)

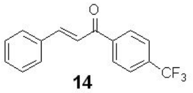
O
CF3

14

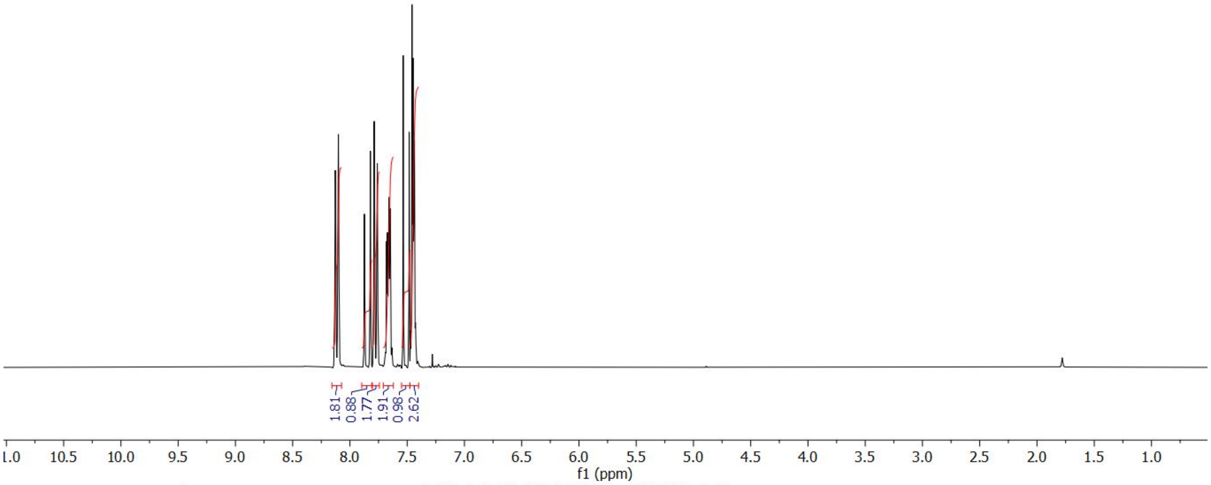
1.81
0.88
1.77
1.91
0.98
2.62
11.0 10.5 10.0 9.5 9.0 8.5 8.0 7.5 7.0 6.5 6.0 5.5 5.0 4.5 4.0 3.5 3.0 2.5 2.0 1.5 1.0
f1 (ppm)

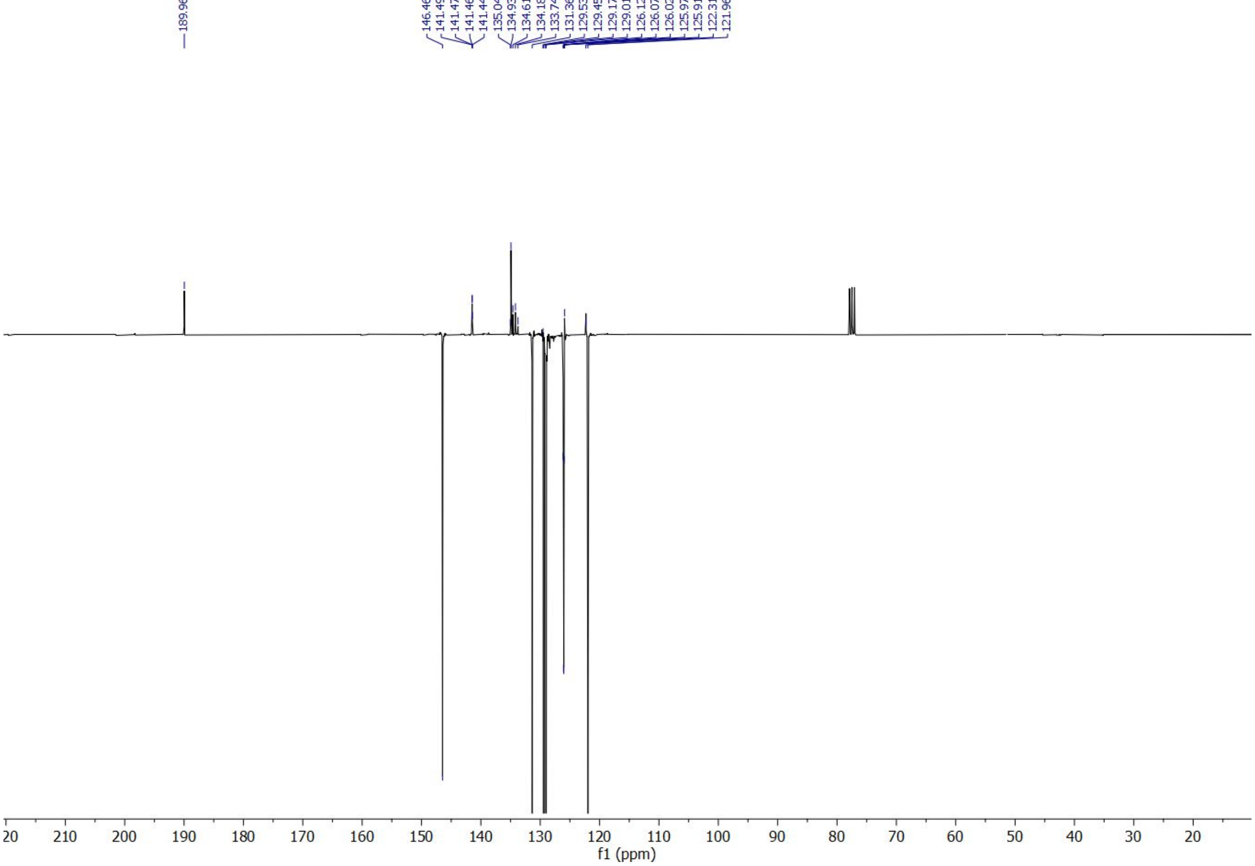
189.96
146.46
141.49
141.47
141.46
141.44
135.04
134.93
134.61
134.18
133.74
131.36
129.53
129.45
129.17
129.01
126.12
126.07
126.02
125.97
125.91
122.31
121.96
220 210 200 190 180 170 160 150 140 130 120 110 100 90 80 70 60 50 40 30 20
f1 (ppm)

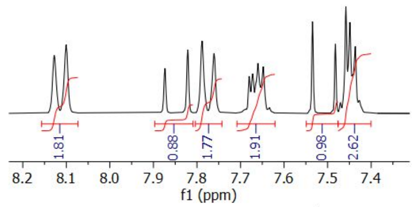
1.81
0.88
1.77
1.91
0.98
2.62
f1 (ppm)

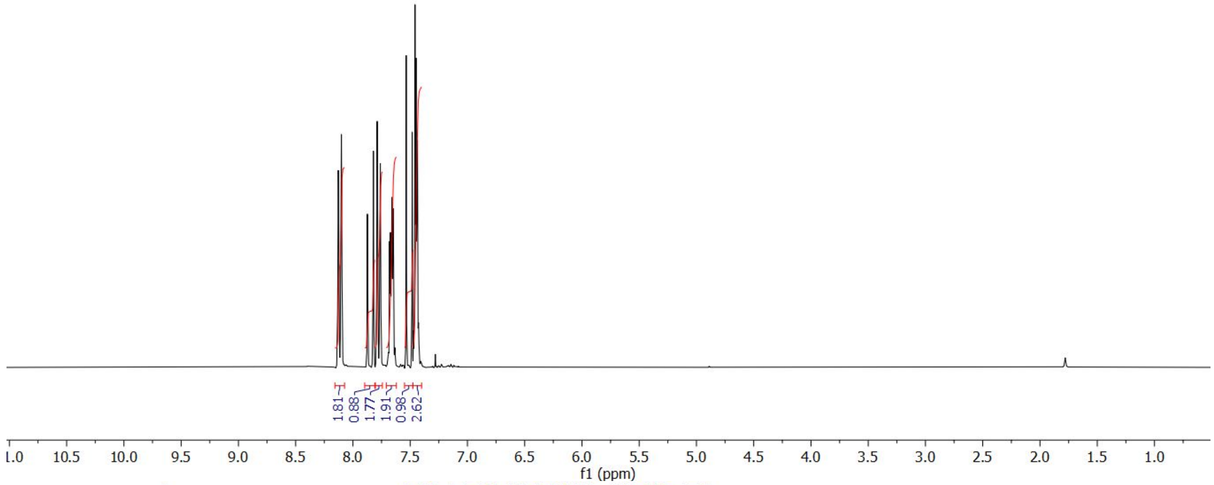
1.81
0.88
1.77
1.91
0.98
2.62
f1 (ppm)

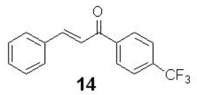
O
CF_3

14

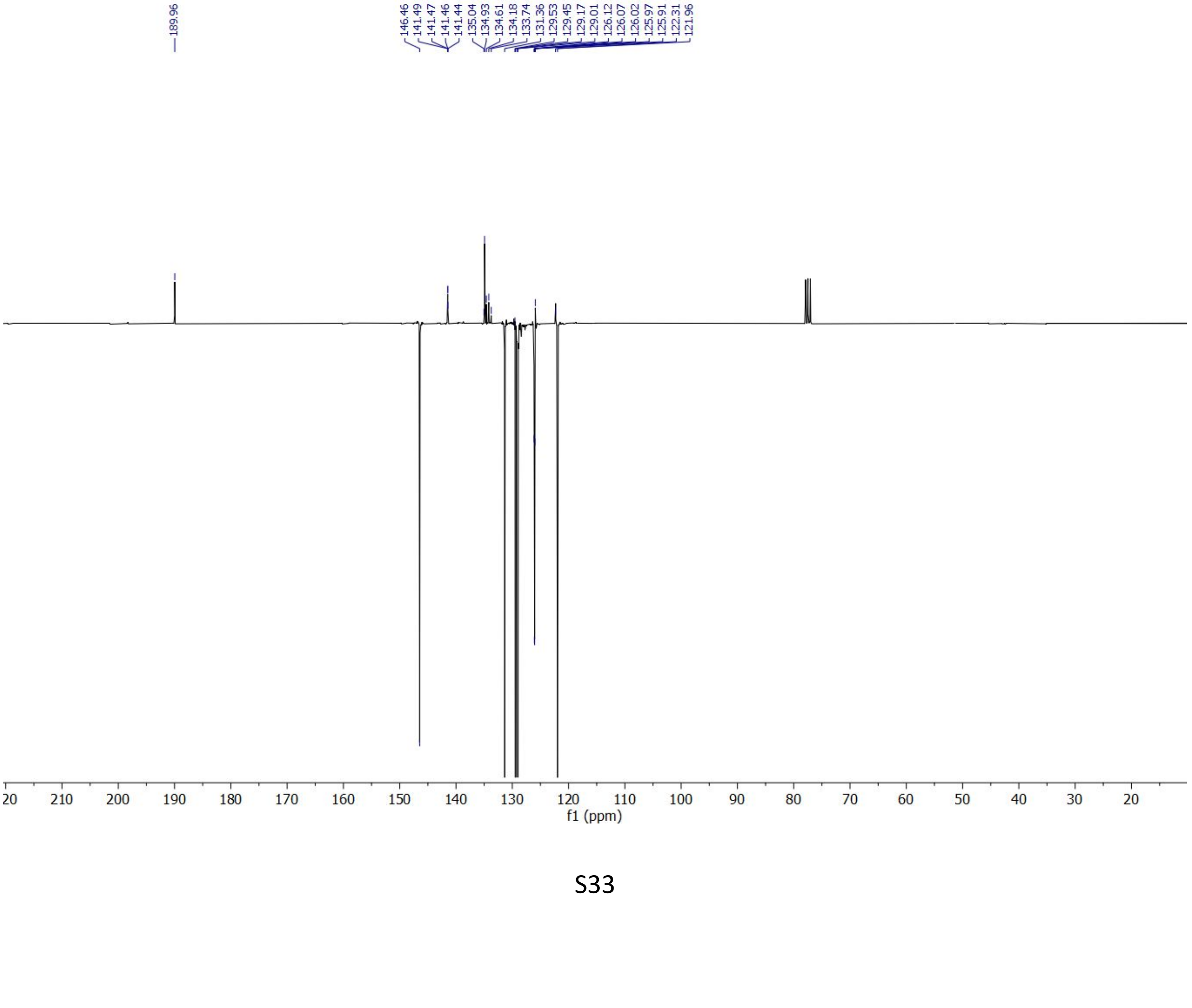
189.96
146.46
141.49
141.47
141.46
141.44
135.04
134.93
134.61
134.18
133.74
131.36
129.53
129.45
129.17
129.01
126.12
126.07
126.02
125.97
125.91
122.31
121.96
f1 (ppm)

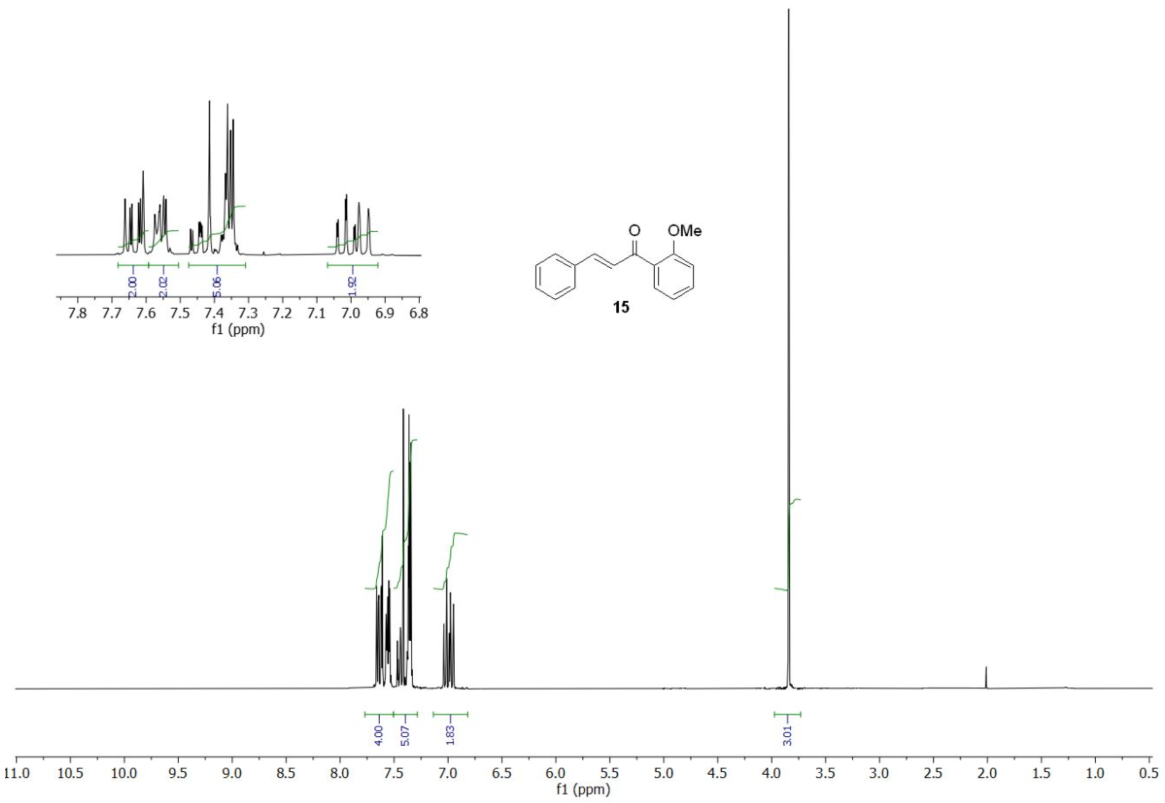
O
OMe
15
2.00
2.02
5.06
1.92
7.8 7.7 7.6 7.5 7.4 7.3 7.2 7.1 7.0 6.9 6.8
f1 (ppm)
4.00
5.07
1.83
3.01
11.0 10.5 10.0 9.5 9.0 8.5 8.0 7.5 7.0 6.5 6.0 5.5 5.0 4.5 4.0 3.5 3.0 2.5 2.0 1.5 1.0 0.5
f1 (ppm)

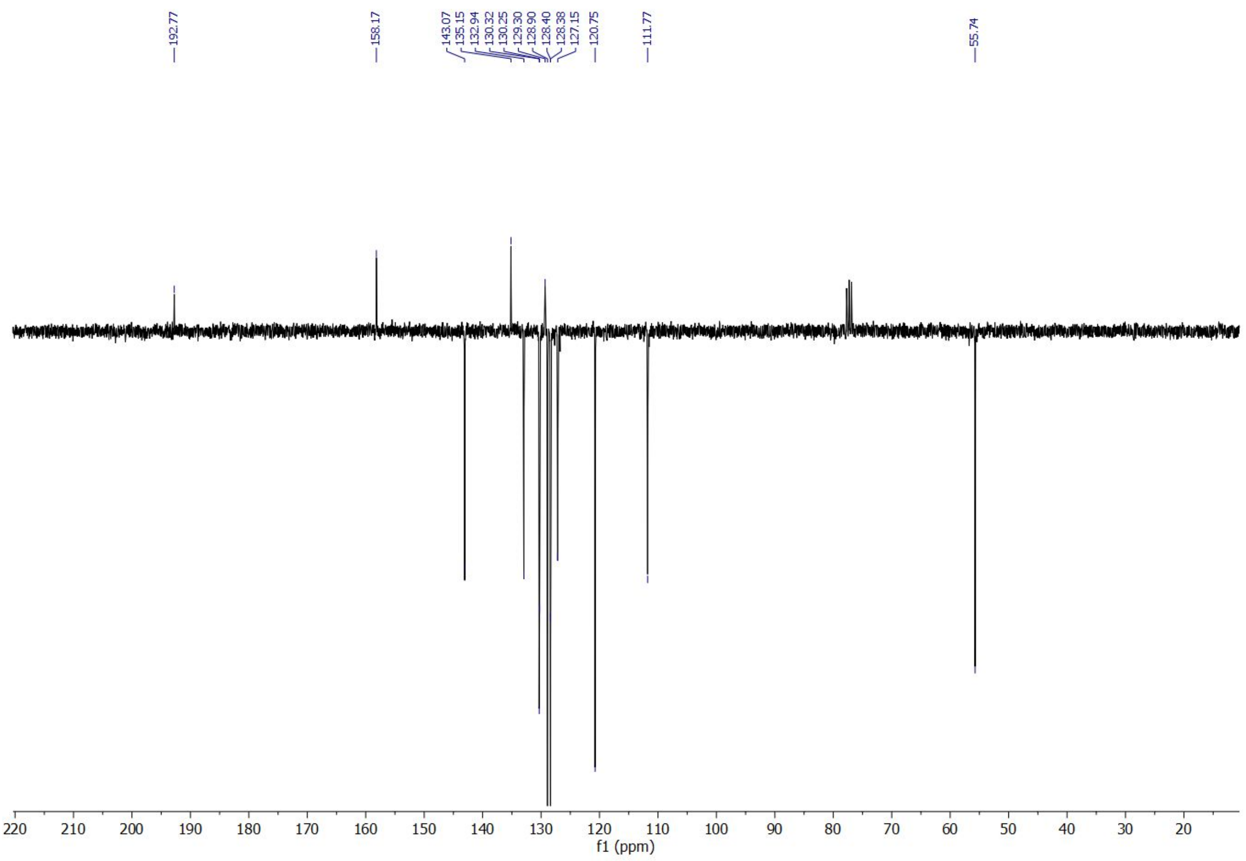
192.77
158.17
143.07
135.15
132.94
130.32
130.25
129.30
128.90
128.40
128.38
127.15
120.75
111.77
55.74
220 210 200 190 180 170 160 150 140 130 120 110 100 90 80 70 60 50 40 30 20
f1 (ppm)

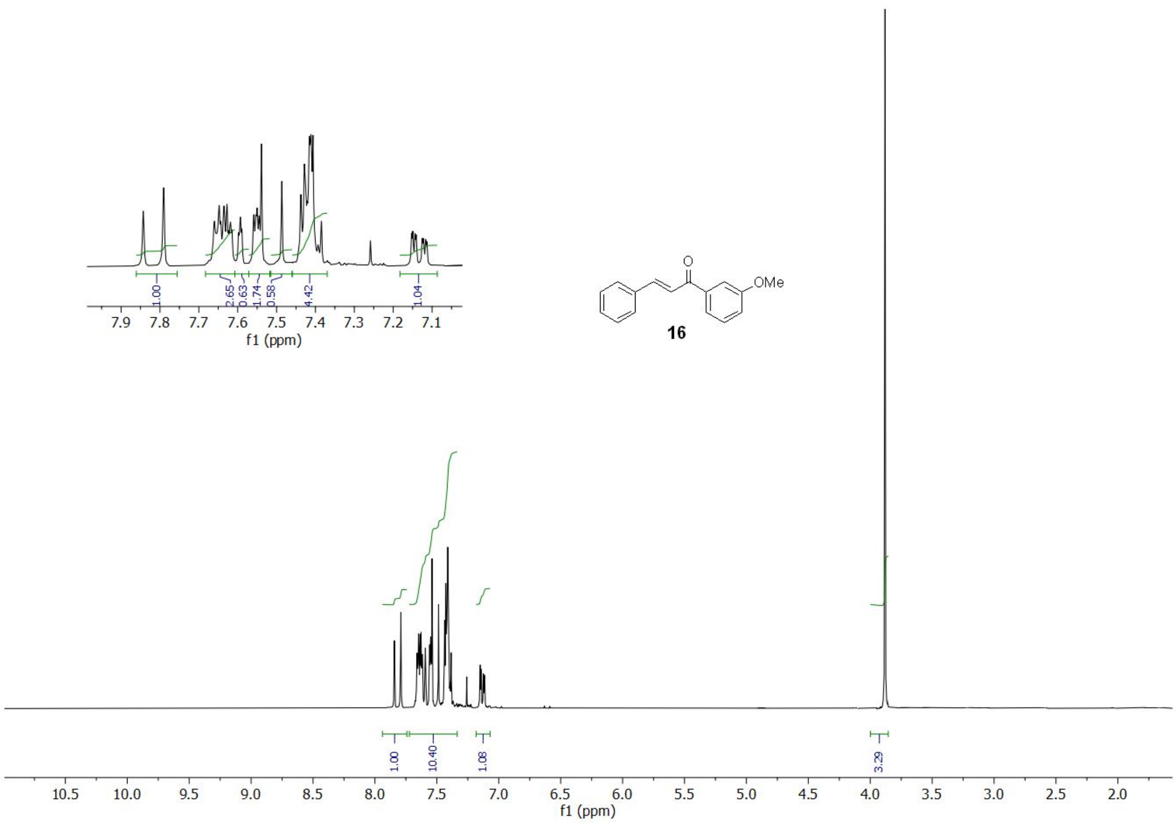
1.00
2.65
0.63
1.74
0.58
4.42
1.04
7.9 7.8 7.7 7.6 7.5 7.4 7.3 7.2 7.1
f1 (ppm)
1.00
10.40
1.08
3.29
10.5 10.0 9.5 9.0 8.5 8.0 7.5 7.0 6.5 6.0 5.5 5.0 4.5 4.0 3.5 3.0 2.5 2.0
f1 (ppm)
O
OMe

16

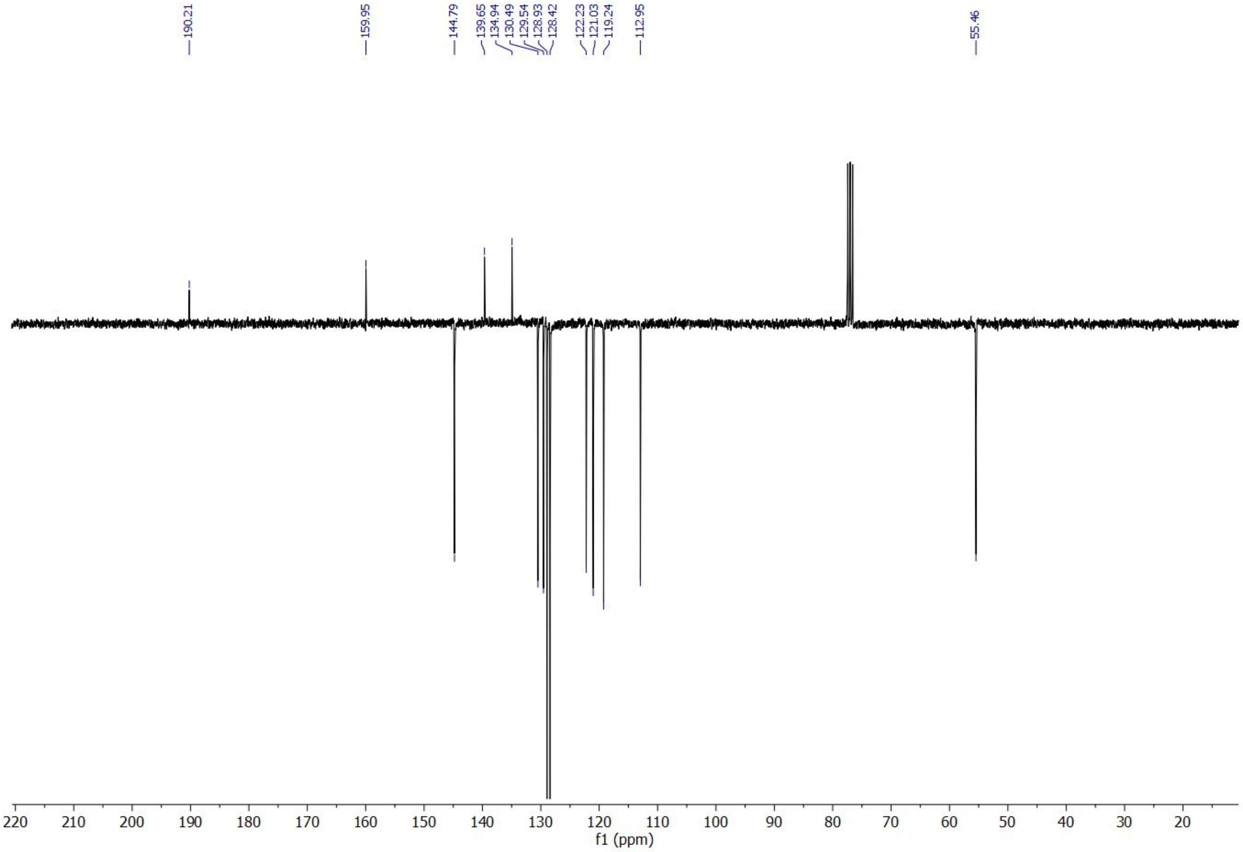
190.21
159.95
144.79
139.65
134.94
130.49
129.54
128.93
128.42
122.23
121.03
119.24
112.95
55.46
220 210 200 190 180 170 160 150 140 130 120 110 100 90 80 70 60 50 40 30 20
f1 (ppm)

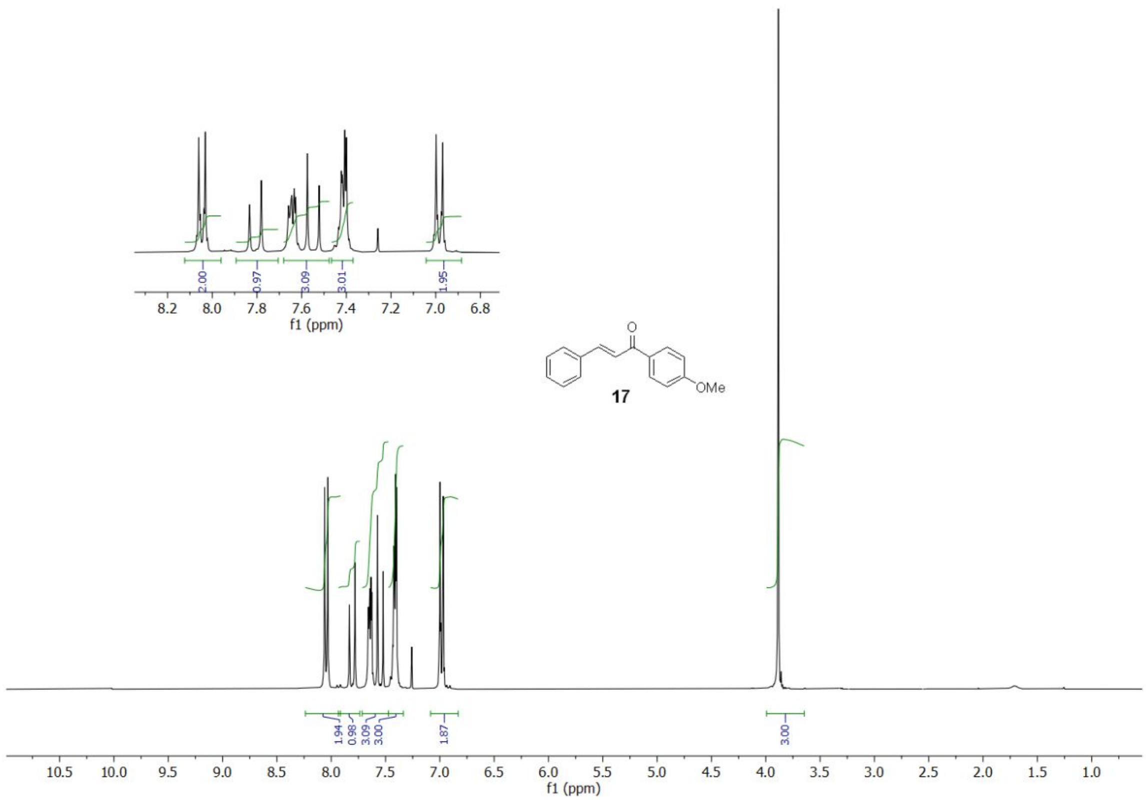
OMe
17
2.00
0.97
3.09
3.01
1.95
8.2 8.0 7.8 7.6 7.4 7.2 7.0 6.8
f1 (ppm)
1.94
0.98
3.09
3.00
1.87
3.00
10.5 10.0 9.5 9.0 8.5 8.0 7.5 7.0 6.5 6.0 5.5 5.0 4.5 4.0 3.5 3.0 2.5 2.0 1.5 1.0
f1 (ppm)

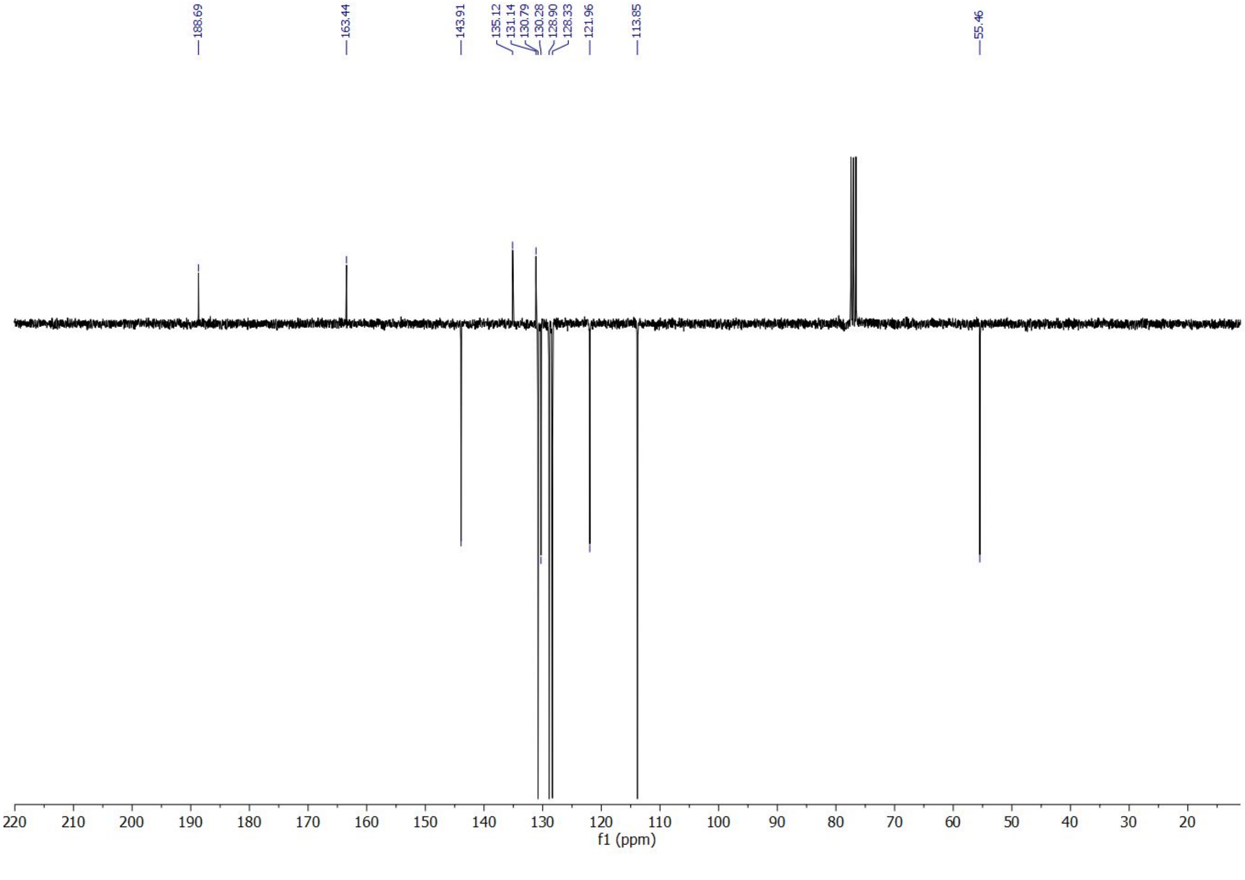
188.69
163.44
143.91
135.12
131.14
130.79
130.28
128.90
128.33
121.96
113.85
55.46
220 210 200 190 180 170 160 150 140 130 120 110 100 90 80 70 60 50 40 30 20
f1 (ppm)
